# Recent Advances in Our Understanding of Age-Related Macular Degeneration: Mitochondrial Dysfunction, Redox Signaling, and the Complement System

**DOI:** 10.14336/AD.2024.0124

**Published:** 2025-01-24

**Authors:** Francesco Buonfiglio, Christina A. Korb, Bernhard Stoffelns, Norbert Pfeiffer, Adrian Gericke

**Affiliations:** Department of Ophthalmology, University Medical Center of the Johannes Gutenberg University Mainz, Langenbeckstr. 1, 55131 Mainz, Germany

**Keywords:** age-related macular degeneration, aging, mitochondrial dysfunction, oxidative stress, complement system

## Abstract

Age-related macular degeneration (AMD) is a prevalent degenerative disorder of the central retina, which holds global significance as the fourth leading cause of blindness. The condition is characterized by a multifaceted pathophysiology that involves aging, oxidative stress, inflammation, vascular dysfunction, and complement activation. The complex interplay of these factors contributes to the initiation and progression of AMD. Current treatments primarily address choroidal neovascularization (CNV) in neovascular AMD. However, the approval of novel drug therapies for the atrophic and more gradual variant, known as geographic atrophy (GA), has recently occurred. In light of the substantial impact of AMD on affected individuals' quality of life and the strain it places on healthcare systems, there is a pressing need for innovative medications. This paper aims to provide an updated and comprehensive overview of advancements in our understanding of the etiopathogenesis of AMD. Special attention will be given to the influence of aging and altered redox status on mitochondrial dynamics, cell death pathways, and the intricate interplay between oxidative stress and the complement system, specifically in the context of GA. Additionally, this review will shed light on newly approved therapies and explore emerging alternative treatment strategies in the field. The objective is to contribute to the ongoing dialogue surrounding AMD, offering insights into the latest developments that may pave the way for more effective management and intervention approaches.

## 1. Introduction

Age-related macular degeneration (AMD) is a highly prevalent and progressive degenerative disorder primarily affecting the central retina, particularly the macular region, encompassing the photoreceptor and retinal pigment epithelium (RPE) layers, Bruch's membrane (BM), and choriocapillaris [1]. As the fourth leading global cause of irreversible visual impairment in individuals aged 50 and above, AMD contributed to 1.8 million cases out of a total of 33.6 million blind adults in 2020 [2]. The principal risk factor for AMD is advancing age [3], and with the worldwide aging population steadily growing, the number of AMD cases is projected to rise from approximately 200 million in 2020 to nearly 300 million by 2040 [4]. Given its widespread prevalence, the economic repercussions of AMD are substantial, posing a significant societal and healthcare challenge [3, 5]. Worldwide direct costs due to AMD-related visual impairment have been estimated at approximatively €343 billion [6, 7]. An observational study in Spain calculated an annual mean direct healthcare cost of over €4,000 per patient [8]. Factoring in indirect costs arising from visual impairment, such as falls, fractures, depression, and reduced quality of life, a recent systematic review revealed that the total annual economic burden per patient, encompassing both ophthalmic and non-ophthalmic expenses, ranges from $7,721 (purchasing power parity) in the UK to $38,665 in the USA [9-11]. Indirect costs from the atrophic variant of AMD, geographic atrophy (GA), reach a significant $24.4 billion yearly in the USA [12, 13].

To address the complex challenges associated with the severe consequences of this disabling disease, its detrimental impact on patients' quality of life, and the substantial costs involved, there is an urgent need for innovative medications [14-16]. Current research efforts are concentrated on gaining a more comprehensive understanding of the primary pathomechanisms underlying AMD onset. The goal is to identify innovative and effective therapeutic targets and design curative strategies that can significantly improve patient outcomes. As a promising outcome of these dedicated endeavors, the U.S. Food and Drug Administration (FDA) has recently approved two complement inhibitors, pegcetacoplan (SYFOVRE®, Apellis Pharmaceuticals, USA) in February 2023 and avacincaptad pegol (IZERVAY®, Astellas Pharmaceuticals, Japan) in August 2023 [17].

**Figure 1.**
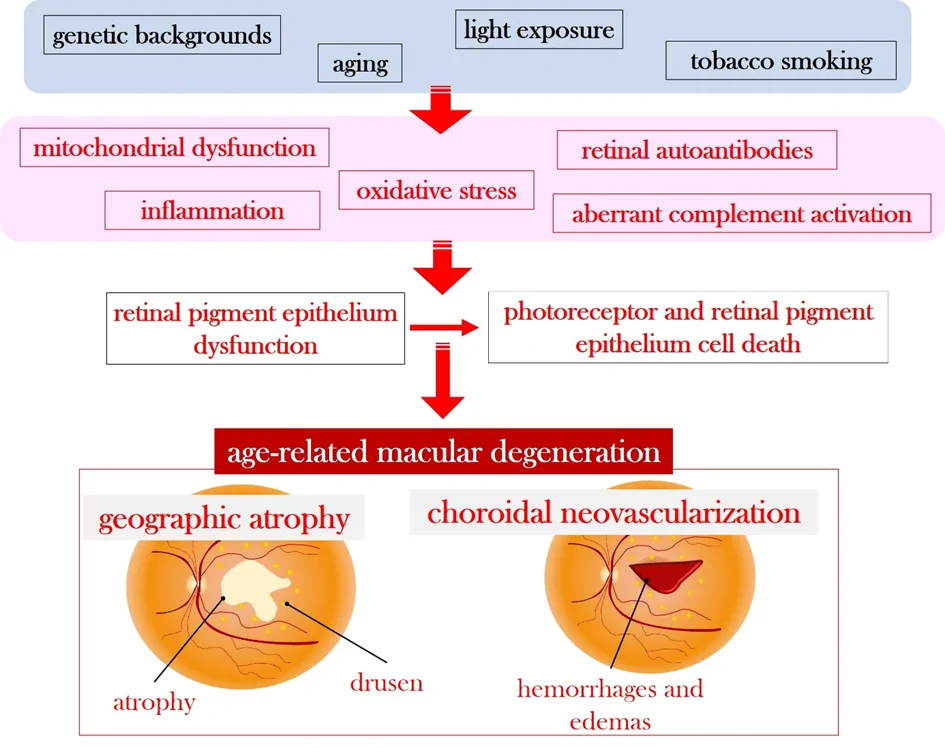
**The multifaceted nature of age-related macular degeneration involves crucial etiopathogenetic factors, including light exposure, aging, tobacco smoking, and genetics**. These factors intricately intertwine, collectively giving rise to pathophysiological changes in the retinal pigment epithelium (RPE) and photoreceptors. This complex interplay manifests through events such as oxidative stress, mitochondrial dysfunction, inflammatory processes, generation of retinal autoantibodies, and aberrant complement activation, ultimately triggering various programmed cell death pathways. The onset of the disease typically becomes apparent in its late manifestation, presenting in two primary forms. The first, geographic atrophy, is the most prevalent variant, characterized by a slow progression and primarily associated with the loss of photoreceptors and RPE cells. The second, choroidal neovascularization, represents a more aggressive variant marked by neoangiogenesis, hemorrhages, and edemas.

In this context, our review article aims to provide a concise summary of the advances in our understanding of AMD's etiopathogenesis, with a particular emphasis on the role of redox pathomechanisms and aging on mitochondria, cell death pathways and complement activation and their interplay in the development and progression of the disorder. Additionally, we will review the latest approved therapeutic approaches and shed light on alternative drug candidates (Fig. 1).

## 2. General Concepts in AMD

### 2.1. Macular Physiology and Retinal Phenotype Characteristics

The retina stands out as one of the most metabolically active organs in the human body, rendering it highly susceptible to oxidative stress emerging as a by-product of physiological processes [18, 19]. The macula, the central region of the retina, spans an oval-shaped area approximately 5-6 mm in diameter [20]. At its core lies the fovea, a depression measuring around 1-1.5 mm in diameter [21]. The central foveal region, spanning roughly 250-600 µm, is devoid of blood vessels and is recognized as the foveal avascular zone [21]. Nutrient and oxygen supply to this area is facilitated through the choroidal microcirculation [21, 22]. Remarkably, the foveal avascular zone harbors the highest concentration of cones in the retina, numbering around 150,000-180,000 cones/mm^2^. In contrast, cone density gradually decreases from approximately 6,000 cones/mm^2^ at a distance of 1.5 mm from the fovea to about 2,500 cones/mm^2^ near the ora serrata [23-26]. The cones in the foveal avascular zone are uniquely narrow and elongated, enabling their extreme centralization, a crucial factor for high-acuity vision [21]. Given the exceptionally high cone concentration and the resulting elevated metabolic and photochemical activity, this region has a substantial oxygen demand, subjecting the retinal pigment epithelium (RPE) to chronic exposure to oxygen partial pressures of roughly 70-90 mmHg [27, 28]. Moreover, the metabolic activities of the RPE, such as phagocytosis, generate an elevated concentration of reactive oxygen species (ROS) [29]. Furthermore, light exposure in the fovea, driven by constant macular light absorption to optimize vision, results in significant photo-oxidative stress [30, 31]. Consequently, numerous sources of ROS in the fovea and its resident RPE significantly impact the susceptibility of the RPE to oxidative stress, a critical contributor to the development of AMD [32-34]. Another population of cells highly relevant in AMD, characterized by a considerable metabolic demand and high mitochondrial activity, is the retinal ganglion cell (RGC) [19]. The axons of RGCs collectively form the optic nerve, with certain portions of these axons being unmyelinated [35]. In these unmyelinated regions, signal transmission cannot occur through saltatory conduction due to the absence of myelin. To compensate for this limitation, RGCs produce elevated amounts of ATP in their axons to repolarize the plasma membrane, facilitated by mitochondrial axonal bi-directional transport [35-37]. Consequently, similar to RPE cells, RGCs have the potential to generate a significant amount of ROS due to their high metabolic activity, making them particularly susceptible to oxidative stress. Moreover, in the myelinated axon portions of RGCs, myelin plays a crucial role as a trophic agent for the optic nerve sheath. Myelin serves to supply nutrients to the axon, supported by evidence indicating the presence of the entire mitochondrial respiratory chain in the myelin sheath of the optic nerve [38]. Reduced mitochondrial respiration associated with myelin may contribute to neurodegenerative processes [39]. Considering that a loss of structural integrity in the myelin sheath occurs during senescence, this event may serve as a trigger for axonal deterioration [40]. Given these insights, it is not surprising that current literature has illuminated pathological AMD-related changes in RGC layers, indicating modified RGC thickness in the context of early or intermediate AMD [41]. A study on RGC layer thickness during intermediate AMD revealed thinning toward the fovea and thickening toward the peripheral macula. Overall, the modifications in RGC layer thickness were more strongly linked with thickening than thinning [42]. Another investigation demonstrated that biological intravitreal therapy has the potential to reverse RGC layer thickening [43]. Collectively, these studies underscore the significance of detecting changes in RGC layer thickness as one of the earliest signs of aging and the onset of AMD.

### 2.2. Risk Factors, Epidemiology, and Genetics

AMD is a multifactorial and complex disease with various factors interacting to disrupting retinal homeostasis, ultimately leading to its onset. Aging is undeniably a key player in this context [3]. Advanced age is associated with a progressive decline in oxidative metabolism, leading to an excess of ROS and chronic oxidative stress, which can cause substantial structural changes and oxidative damage in the retina [44, 45]. Beyond aging, other factors, such as modifiable environmental elements like tobacco smoking, hypercholesterolemia, obesity, arterial hypertension, and cardiovascular diseases, are associated with increased ROS levels and are recognized as risk factors for AMD [46-48]. In addition, light exposure, particularly in the form of UV radiation and blue light, which can induce ROS formation, has been linked to AMD and considered a possible factor in disease onset or progression [49-52]. Moreover, specific chemical substances, such as perfluorooctanoic acid found in indoor particulate matter, have been identified as potential inducers of oxidative stress and may be regarded as risk factors for AMD [53]. The impact of gender on the occurrence of AMD is a subject of debate. While some studies have reported a potential association between female gender and higher progression rates compared to males [54, 55], other studies have found no link between female gender and AMD progression [56-58]. Nonetheless, some attribute the potential gender-related difference in disease progression to the longer life expectancy of females compared to males [59].

Notably, the X-linked gene, DIAPH2 (diaphanous related formin 2), known to be related with premature ovarian failure, has been suggested to be associated with AMD [60, 61]. In addition to gender, the prevalence of AMD varies by ethnicity, with higher rates among Caucasians compared to, for example, Chinese or Africans, and geographic location, with higher rates in Europeans compared to Americans [4, 62-70]. These epidemiological variations suggest the possibility that differences in susceptibility genes for AMD between populations may result in varying occurrences and subtypes of the disorder [62, 71]. In this context, a recent investigation highlighted that genetics and diet may interact to influence the onset of AMD, potentially revealing a protective effect of specific contexts such as the Mediterranean diet and specific genetic profiles [72].

The current literature points to numerous genetic factors as potential contributors to the onset of the disorder [62]. Genome-wide association studies have identified specific gene variants encoding complement factors, particularly complement factor H (CFH), as well as CFI, CFB, C2, C3, and C9, linked to an increased risk of AMD [73-77]. Additionally, strong associations with the disease have been observed for specific polymorphisms in the age-related maculopathy susceptibility 2 (ARMS2) and high-temperature requirement A serine peptidase 1 (HTRA1) genes located on chromosome 10, specifically within the 10q26 locus [78-80]. The role of ARMS2 in the etiopathogenesis of AMD is still under investigation. Initially, it was hypothesized that ARMS2 might encode a protein expressed on the outer mitochondrial membrane in photoreceptors and RPE cells. However, subsequent studies have suggested localization in the cytoplasm or on the endoplasmic reticulum (ER), or even a role as an extracellular matrix component [62, 79]. It has also been proposed that ARMS2 may be involved in local inflammation via release by macrophages, implicating it in the activation of the complement system [81]. Interestingly, a recent study by Chang et al. demonstrated that a specific ARMS2 variant is associated with increased oxidative stress in induced pluripotent stem cell (iPSC)-derived retinal cells from AMD patients [82]. HTRA1 encodes a heat shock protein with serine protease activity, responsible for the degradation of extracellular matrix proteins and the regulation of TGF-β signaling [83]. A study by Beguier and colleagues found that AMD-associated variants of HTRA1 can exacerbate subretinal inflammation, interfering with the normal removal of monocytes [84]. Conversely, a more recent investigation by Williams et al. suggested that HTRA1 is crucial in modulating subretinal inflammation and maintaining the RPE-BM interface during aging, indicating that its upregulation, rather than inhibition, may be a potential therapeutic strategy [85].

Importantly, certain gene variants involved in lipid metabolism have been linked to AMD. This disorder is characterized by significant lipid dysmetabolism, resulting in the abnormal accumulation of lipid byproducts containing apolipoprotein (Apo) B and E [86]. Within this context, specific polymorphisms of the ApoE gene, particularly those related to the Ԑ2 isoform, have been associated with an increased risk of AMD [87].

### 2.3. Staging, Progression, and Clinical Presentation

AMD unfolds as a gradual accumulation of drusen, distinctive markers of early disease stages. Drusen, lipid-rich deposits of extracellular material, typically reside between BM and the RPE in the macular region. Over time, these drusen contribute to degeneration and atrophy affecting the photoreceptor layers, RPE, BM, and choroidal microcirculation [1, 88]. Various classification systems have emerged over the past decades to delineate different phases of AMD, primarily based on drusen presence, size, and potential pigmentary abnormalities [89-91]. Currently, one of the most widely used staging systems is the one developed by Ferris et al. in the context of the Age-Related Eye Disease Study (AREDS) and endorsed by the Beckman Initiative for Macular Research Classification Committee. This system distinguishes five phases based on drusen and pigmentary changes [92]:

1. No AMD: Absence of drusen or pigmentary abnormalities.
2. Normal Aging: Presence of small drusen (≤63 µm).
3. Early AMD: Detectable medium-sized drusen (>63 µm and ≤125 µm).
4. Intermediate AMD: Presence of large drusen (>125 µm) and pigmentary anomalies.
5. Late AMD: Manifests as either the atrophic variant (aAMD or "dry" AMD), accounting for around 80-85% of all advanced stages [93, 94], or the neovascular variant, also known as "wet" AMD or neovascular AMD involving exudation.

**Figure 2.**
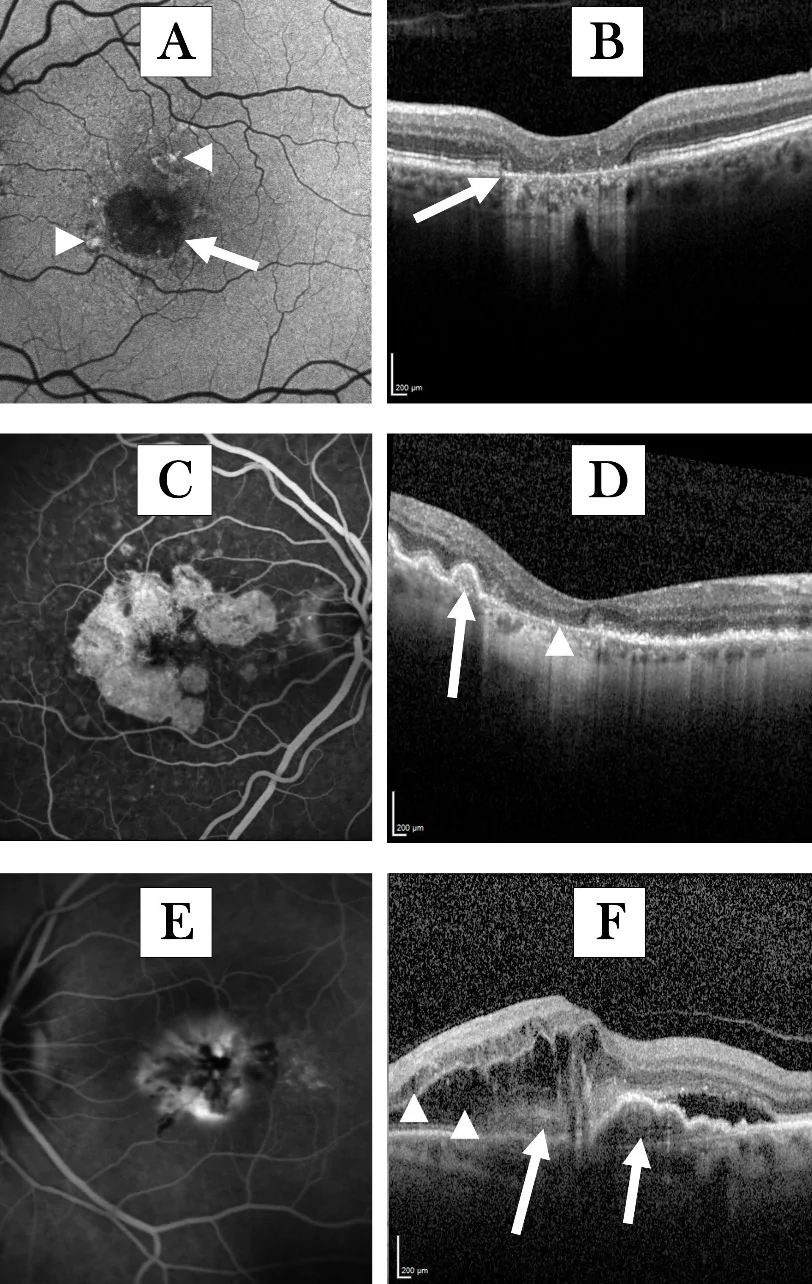
**Fundus autofluorescence, fluorescein angiography photographs, and horizontal OCT scans over the fovea depict examples of geographic atrophy (GA) (A-D) and neovascular age-related macular degeneration (AMD) (E and F)**. In panel (A), a fundus autofluorescence image from a patient with geographic atrophy is presented. The white arrow indicates the atrophic area characterized by a significant reduction in fundus autofluorescence due to the absence of retinal pigment epithelial (RPE) cells, resulting in the absence of autofluorescent lipofuscin. The white arrowheads highlight spots of increased autofluorescence attributed to lipofuscin accumulation and drusen surrounding the atrophic area. (**B**) Utilizing high-resolution spectral-domain OCT (SD-OCT), the image reveals significant thinning of the RPE (indicated by the white arrow) and the loss of the overlying outer retinal layers within the atrophic area. (**C**) In fluorescein angiography, atrophic patches manifest as clearly defined, brightly fluorescent areas. This occurs due to heightened visibility of the underlying choroidal fluorescence resulting from the absence of RPE cells. Normally, these cells would attenuate fluorescein transmission. (**D**) With SD-OCT, an irregular appearance of the RPE band with dome-shaped elevations of the RPE corresponding to drusen (white arrow), RPE thinning (white arrowhead), and loss of outer retinal layers is seen. (**E**) Fluorescein angiogram illustrating a classic choroidal neovascularization (CNV) characterized by abnormal leakage in regions of choroidal neovascular membranes. (**F**) The OCT scan shows an elevated and disrupted RPE, hyperreflective material (white arrows) below and above the RPE, as well as sub-and intraretinal fluid (white arrowheads).

Figure 2 presents clinical examples of GA and neovascular AMD obtained by fluorescein angiography and optical coherence tomography (OCT).

While the majority of patients with AMD experience the same disease phase in both eyes, in cases of unilateral involvement, the second eye becomes affected within approximately five years in nearly one-third of cases [95, 96]. The likelihood of progression to advanced AMD from intermediate stages is estimated at around 28% over five years [97]. Significantly, multiple studies underscore the substantial influence of the fellow eye's health on disease progression. Notably, a recent extensive cohort study in the UK investigated the impact of the fellow eye on progression to late stages. The findings revealed that eyes with early/intermediate AMD and a diagnosis of choroidal neovascularization (CNV) in the partner eye progressed to CNV more rapidly. Similarly, those with a diagnosis of geographic atrophy (GA) in the fellow eye exhibited a swifter progression compared to other subgroups [98].

Early AMD is usually asymptomatic and may progress to intermediate or late AMD without noticeable symptoms [99, 100]. Patients typically report their first symptoms when the disease has advanced, often experiencing a sudden decrease in visual acuity uncorrectable with refractive adjustments and distortions in the central visual field, (metamorphopsia), detectable through the use of an Amsler grid [100]. Following a thorough fundus examination, one of the most valuable diagnostic tools in clinical practice is the OCT of the macular region. OCT reveals the size, location, distribution, and quantity of drusen, detecting intraretinal cystic fluid or subretinal fluid [63]. OCT plays a pivotal role in AMD management, serving for primary diagnosis, clinical assessment, monitoring medication effectiveness, and long-term tracking of disease progression [101]. Various diagnostic tools aid in detecting or characterizing the stage of AMD, including microperimetry [102], optical coherence tomography angiography (OCTA) [103, 104], as well as fluorescein angiography (FA) and indocyanine green angiography (ICGA). FA and ICGA are critical for detecting CNV and typical regions of leakage resulting from angiogenic processes [63, 67, 105]. Newly formed vessels can rupture, leading to significant hemorrhages and the formation of dysfunctional macular scarring [100]. While GA progresses slowly, gradually affecting visual acuity, CNV follows a more aggressive disease course. Despite accounting for approximately 10-20% of all AMD cases, CNV is responsible for 80-90% of AMD-induced blindness [105].

### 2.4. Composition and Phenotypes of Drusen

Drusen plays a pivotal role in the clinical diagnosis of AMD, necessitating a thorough understanding of their structural characteristics and various phenotypes. Structurally, drusen primarily consists of lipid accumulation and carbohydrates depositing within Bruch's membrane (BM) during the aging process. This deposition contributes to a gradual thickening and impairment of BM, ultimately leading to AMD [106]. However, this event appears to be a consequence of the activity of RPE cells. RPE cells regulate the elimination of metabolites produced by photoreceptors and release lipoproteins containing apolipoprotein (Apo) B and E as byproducts of lipid metabolism [63, 107]. Age-related oxidative stress-induced impairment of the RPE may result in dysfunctional degradation of these metabolites, a central pathogenic event in AMD that leads to drusen formation [107-111]. The expansion and increasing deposition of drusen along BM can be critical, disrupting this membrane, a vital connection between the RPE and choroid, responsible for the normal supply of oxygen and nutrients. This disruption can ultimately lead to hypoxia and neoangiogenesis, culminating in the loss of RPE and photoreceptors, tissue degeneration, atrophy, and choroidal vascular dysfunction [109, 112, 113].

Importantly, several complement proteins, including C1q, CFH, C5, C6, C8, and C9, have been found within drusen. This discovery offers insights into potential molecular targets for AMD treatment [110, 114-116]. The presence of complement components in drusen may be attributed to impaired RPE clearance, leading to the release of complement proteins alongside lipids, or the vulnerability of the choriocapillaris to immunocomplex deposition, potentially activating the complement system [115, 117, 118].

Traditionally, drusen have been categorized into two subgroups based on their appearance during fundus evaluation: "hard" and "soft" drusen. "Hard" drusen typically appear as punctate yellow nodules and may signal a subsequent development of atrophy. However, "hard" drusen can also spontaneously regress without progressing to GA [119]. “Soft” drusen manifest as large, typically over 63 µm in diameter, light-yellow or grayish-white, dome-shaped elevations preceding the development of clinically evident CNV [119]. With the introduction of OCT in clinical practice, various drusen phenotypes have been defined based on their size, shape, and reflectivity, including large colloid drusen, pachydrusen, and cuticular drusen [120].

Remarkably, individuals carrying the ARMS2/HTRA1 risk haplotype have been shown to exhibit a considerable susceptibility to the progression of GA, CNV, reticular pseudodrusen (RPD), and large drusen [121-123]. RPD, also known as subretinal drusenoid deposits, differ from conventional drusen as they are located above, rather than below, the RPE level [124]. According to findings by Thee and colleagues, RPD exhibit a robust association with the ARMS2/HTRA1 risk haplotype, highlighting that carriers of this risk haplotype have a sixfold higher risk of presenting RPD compared to noncarriers of the risk variant [125].

Phenotyping drusen can be essential to differentiate diverse pathogenetic pathways and their varying impact on AMD progression, potentially leading to more personalized treatment strategies [126]. For example, RPDs have been associated with a higher risk of disease progression and have recently been linked to coexisting high-risk vascular diseases such as myocardial infarctions or strokes [127]. However, the presence of RPDs has also been demonstrated in other conditions, such as macular dystrophies or macular atrophies with pseudodrusen-like appearances [128, 129]. Apart from RPDs, other high-risk prognostic biomarkers include large-volume drusen and the presence of hyper-reflective loci [130, 131]. Conversely, cuticular drusen have been shown not to be associated with a higher risk of AMD progression [120]. Additionally, the existence of pachydrusen, a relatively recent phenotype characterized by drusen with irregular shapes associated with a thicker choroid and a broader distribution at the posterior pole, has been noted to be linked with a specific type of choroidal neovascularization (CNV), namely, polypoidal choroidal vasculopathy [132-135].

### 2.5. Prevention, Established Treatments, and Prognosis

The management of AMD underscores the importance of addressing modifiable risk factors, particularly tobacco smoking, given their significant impact on patient outcomes after treatment [100, 136].

Dietary supplementation with substances like lutein or omega-3 fatty acids has demonstrated some beneficial effects in intermediate AMD, though not in the early or late stages [137]. Therefore, these options are not suitable for prophylactic treatment until intermediate AMD is diagnosed. However, adopting a typical Mediterranean diet, rich in fruits, vegetables, legumes, and fish, has been proven effective for primary prevention and is highly recommended [138]. A debated curative option for intermediate stages involves nanosecond laser therapy on drusen. However, a dedicated trial did not demonstrate benefits in slowing the progression of AMD or providing protective effects. In the case of RPDs, nanosecond laser treatment even accelerated disease progression [139].

The recent approvals for the treatment of GA are discussed in detail later (section 4.1). As for therapeutic options for the exudative and more aggressive variant, CNV, the past two decades have seen significant advancements through the introduction of biologics or monoclonal antibodies with VEGF-inhibitory activity targeting VEGF or its receptor [140]. Before the introduction of anti-VEGF agents, initial treatment attempts for CNV, including laser photocoagulation and photodynamic therapy, were possibly effective in preventing severe visual acuity loss but did not improve visual outcomes [140]. Anti-VEGF agents have revolutionized the field of treatment possibilities and visual prognosis in AMD, offering benefits in terms of improved visual acuity and becoming the gold-standard therapy for CNV [141, 142]. Four intravitreally administered anti-VEGF medications are currently available: bevacizumab, utilized off-label since 2005; ranibizumab, officially licensed in 2006 in the US and in 2007 in Europe; aflibercept, granted approval in 2011 in USA and in 2012 in Europe; brolucizumab, more recently approved in 2019 in USA and in 2020 in Europe [100]. Additionally, conbercept, another anti-VEGF agent known for its high affinity for both isoforms of VEGF-A and VEGF-B similar to aflibercept, has been authorized for the treatment of choroidal neovascularization (CNV) in China since 2013 and in Mongolia since 2020 [143]. A more recent addition is faricimab, a novel monoclonal antibody targeting both VEGF and angiopoietin-2, which received approval from the FDA and European Medicines Agency (EMA) in 2022 for the treatment of CNV and diabetic macular edema [144].

**Figure 3.**
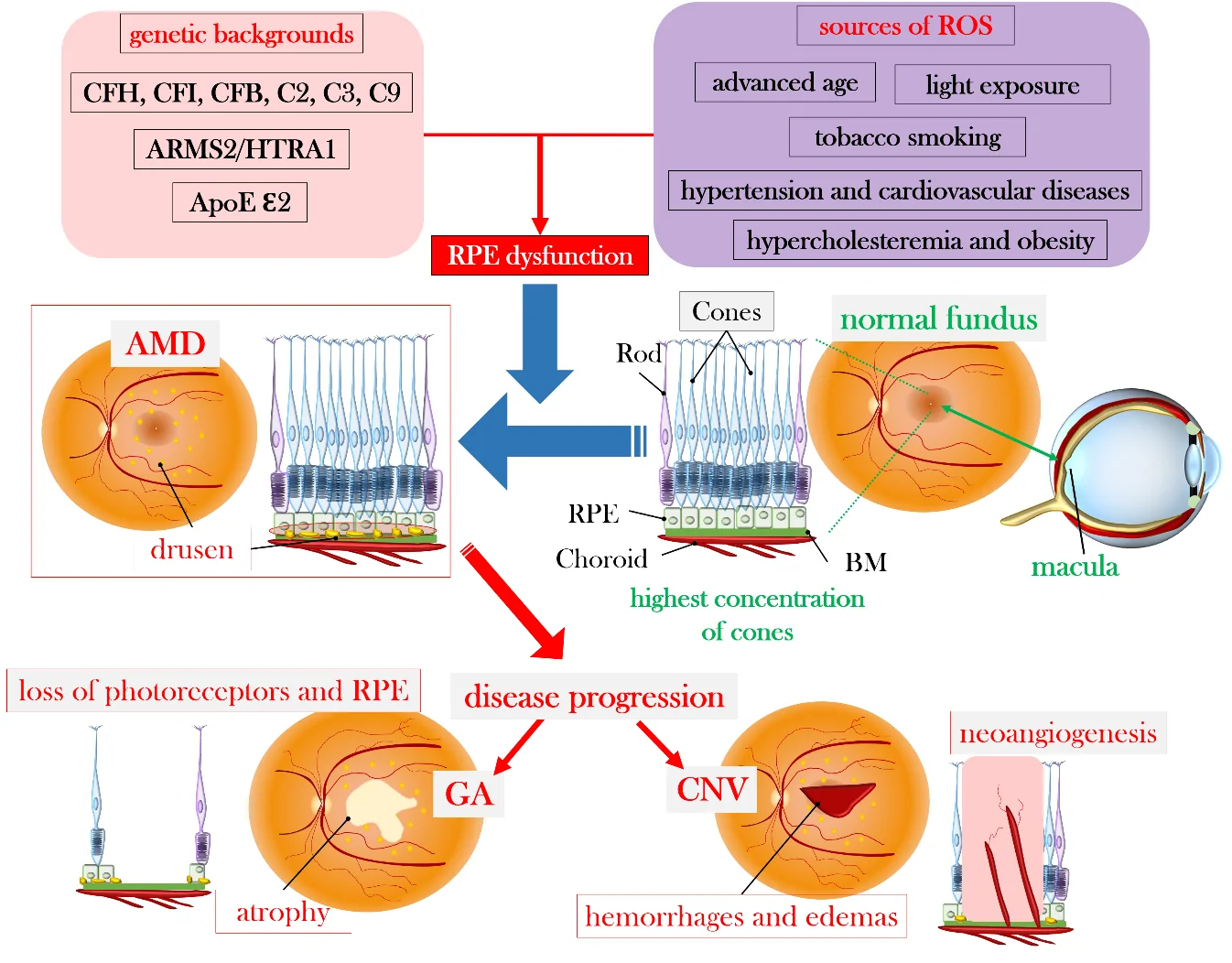
**Illustration on risk determinants and presentation in AMD**. CF: complement factor; ROS: reactive oxygen species; RPE: retinal pigment epithelium; AMD: age-related macular degeneration; ARMS2: age-related maculopathy susceptibility 2 gene; HTRA1: high-temperature requirement A serine peptidase 1 gene; ApoE: apolipoprotein E; BM: Bruch’s membrane; GA: geographic atrophy; CNV: choroidal neovascularization.

Importantly, anti-VEGF therapy is effective when continuously administrated over the long-term, typically at least in the first few years of treatment, to reduce the risk of vision loss [145]. Different dosing protocols have been developed, including the pro re nata or treat-and-extend schedules [100, 140]. Nevertheless, in most cases, approximately 7-8 administrations in the first year of therapy are required to effectively combat CNV development [146]. Consequently, the long-term persistence of therapy, with short intervals between injections, can pose a significant burden on patients and their families [147], raising concerns about consistent patient adherence to treatment. Of note, studies have also indicated that anti-VEGF treatment in patients with CNV may potentially contribute to the development of GA [148, 149]. For example, the "Comparison of Age-related Macular Degeneration Treatments Trials" (CATT) research group observed that among the 1011 participants who were not affected by GA at baseline and were undergoing anti-VEGF therapy, there was a cumulative incidence of GA of 12% at one year, 17% after two years, and 38% at five years [150].

The prognosis for CNV has significantly improved with the introduction of anti-VEGF therapy. The reduced increase in blindness rates for AMD, despite its growing prevalence due to an aging population, can likely be attributed to the introduction of anti-VEGF treatments against CNV [100]. Nevertheless, among eyes with intermediate AMD, GA still develops in 19% within 5 years [151]. Although this variant evolves more slowly, the need for new drugs remains crucial to improve visual outcomes for this patient population.

Figure 3 summarizes risk determinants, macular structural changes and clinical manifestations in AMD.

## 3. Current Understanding of AMD Pathophysiology

### 3.1. Retinal Pigment Epithelium and Oxidative Stress

#### 3.1.1. Susceptibility to Oxidative Stress

The RPE constitutes a postmitotic polarized single layer of cells situated between photoreceptors and the BM. Structurally, its inner side, or apical surface, facing the photoreceptors, presents long microvillous prominences that functionally interact with the photoreceptor outer segment (POS) of cones and rods [152]. On its outer part, or basal surface, directed towards the BM, the RPE is characterized by a surface with small infoldings, augmenting the cell surface to facilitate increased substance exchange [152]. The RPE plays numerous fundamental roles in the retina. It is responsible for maintaining retinal homeostasis, modulating the flow of blood-derived nutrients and growth factors to the POS, and constituting the outer blood-retinal barrier [153, 154]. In addition, the RPE is vital for physiological visual activity, regenerating 11-cis-retinol from all-trans-retinol during the retinoid cycle and facilitating its return to the photoreceptors [155]. Moreover, the RPE is essential in modulating retinal immune responses by releasing various cytokines, such as IL-1β and TNF-α [156, 157], and in regulating ion membrane transport in the subretinal space [158, 159]. Most importantly, the RPE maintains photoreceptor functionality through processes of POS phagocytosis, enabling the physiological turnover of POS with approximately 30,000 daily ingested disk segments [154, 160, 161]. Noteworthy is the RPE's role in protecting photoreceptors from chronic oxidative stress exposure through the clearance of POS-contained free radicals and photodamaged proteins [162, 163]. POS, in turn, incorporates a substantial amount of polyunsaturated fatty acids (PUFAs), which are oxidized by the RPE through NOX and peroxisomes, generating ROS during phagocytosis [163-166]. Given its multiple activities, the RPE exhibits a high metabolic demand, reflected in an elevated number of mitochondria [154, 167]. These organelles serve as a pivotal source of energetic biomolecules, such as adenosine triphosphate (ATP), obtained from the electron transport chain (ETC), which, despite collaterally inducing the formation of ROS in physiological circumstances, significantly increases in cases of mitochondrial impairment. This relationship between ROS and mitochondrial DNA damages aligns with the theory of the "mitochondrial vicious cycle of aging" [165, 168, 169]. Notably, mitochondrial oxidative stress in the RPE contributes to metabolic dysfunction in both RPE and photoreceptors [170]. Considering the high oxygen exposure, metabolic demand, and phagocytosis-related ROS formation, the RPE stands out as particularly prone to oxidative stress and represents one of the most significant sources of ROS in the retina [154, 162, 171].

#### 3.1.2. Sources of ROS: Light Exposure and Tobacco Smoking

In addition to phagocytic processes, UV radiation and blue light contribute to an overabundance of ROS in RPE cells, creating a condition known as photooxidative stress [52]. In an *in vitro* cell-based model, exposure to UVB light resulted in a dose-dependent decrease in cell viability, accompanied by an increase in ROS concentrations. The study also revealed an upregulation of the apoptotic marker Bax, and a downregulation of the antiapoptotic marker Bcl-2 [172]. Furthermore, UV exposure may induce the overexpression of proinflammatory mediators via NF-κB activation and depletion of ATP, potentially in the context of mitochondrial dysfunction. Reduced ATP levels may hinder effective RPE phagocytosis, promoting RPE hyperpigmentation, a recognized risk factor for AMD [30].

Blue light radiation similarly induces ROS excess, primarily through increased NADPH oxidase (NOX) and mitochondria-like activity, particularly in the outer segments of photoreceptors in a cell-based model [173]. Rhodopsin-dependent toxic effects of blue light on the retina have been observed, suggesting that decreased blue light radiation may reduce the risk of AMD [174]. Despite these findings, large epidemiologic studies assessing the impact of blue light-blocking intraocular lenses have not demonstrated clear benefits [175].

Tobacco smoking, like light exposure, stands out as one of the most impactful exogenous risk factors for AMD [176, 177]. In a murine model exposed to tobacco cigarette extracts for 6 months, RPE apoptosis and drusen-like deposits through ROS excess have been observed [30]. Nrf2, a fundamental transcriptional factor that modulates the expression of antioxidative agents, is activated in response to the RPE's reaction to tobacco smoking [178]. Of note, in a Nrf2-deficient mouse model changes similar to those seen inhuman AMD have been observed [179]. Apart from enhancing antioxidative defense, Nrf2 triggers the expression of autophagy genes, such as p62 [180]. Intriguingly, p62 is responsible of protein aggregation processes via its ubiquitin-associated (UBA) domain in autophagy [180]. Mechanistically, in absence of stressors, Nrf2 binds to Kelch-like ECH-associated protein 1 (Keap1) and is later removed in the proteasome. In case of p62 upregulation, a competition to bind Keap1 between Nrf2 and p62 is established, prolonging the activity of Nrf2 and ultimately leading to higher antioxidant capacity. Overall, a crosstalk between Nrf2, p62, and oxidative stress may play a critical role in the etiopathogenesis of AMD, offering the possibility of targeting these molecules to enhance autophagy in RPE and counteract ROS overabundance [181].

#### 3.1.3. Insights into Cell Death Pathways in AMD

One of the most extensively studied cell death pathways associated with mitochondria in AMD is the intrinsic apoptotic pathway. In this cascade, the activation of pro-apoptotic Bcl-2 proteins Bax and Bak is a crucial step that promotes mitochondrial apoptosis [182]. This event leads to the release of cytochrome c, enabling the formation of the apoptosome (formed by caspase 9, Apaf-1 and cytochrome c), followed by the activation of caspase 3, ultimately causing cellular membrane disassembly [183]. Mitochondrial dysfunction, associated with mitochondrial outer membrane permeabilization (MOMP), is linked to inflammatory effects via the release of macromolecules such as fragments of mtDNA or damage-associated molecular patterns (DAMPs) [182]. While apoptosis is generally caspase-mediated, immunotolerant, and immunosuppressive, necrotic programmed events or necroptosis are kinase-mediated and characterized by inflammation, ATP depletion, and disruption of nuclear and cellular membranes. Necroptosis is initiated by stimuli such as TNF-α or lipopolysaccharide (LPS), which subsequently induce the phosphorylation of receptor interacting protein kinase 1 (RIPK1) and RIPK3. This cascade of events leads to the phosphorylation of the mixed lineage kinase domain-like pseudokinase (MLKL). MLKL, upon oligomerization, forms a membrane pore, resulting in cell permeabilization and eventual rupture of the cell membrane [184]. This process is morphologically defined by cell swelling and bursting [185]. Necroptotic cell death, rather than apoptotic, has been detected in RPE cells exposed to high ROS concentrations, potentially related to calcium overload [186, 187]. Lipofuscin has been implicated in causing atypical necroptosis through lysosomal membrane permeabilization in AMD [188]. However, the role of mitochondria and of mitochondrial ROS generation in necroptotic processes remains debated and unclear [162, 189].

Pyroptosis, another form of cell death strongly associated with inflammation, is triggered by various stress stimuli, including elevated mitochondrial ROS, pathogen-associated molecular patterns (PAMPs), damage-associated molecular patterns (DAMPs), and LPS [190-192]. The activation of the NLRP3 (NOD-like receptor, nucleotide-binding domain, leucine-rich-containing family, pyrin domain-containing-3) inflammasome by these stimuli leads to the recruitment of caspase 1 through the adapter protein apoptosis-associated speck-like protein containing a caspase activation and recruitment domain (ASC). Caspase 1 plays a critical role in the cleavage of pro-IL-1β, pro-IL-18, and gasdermin D (GSDMD), facilitating the formation of a cell membrane pore upon oligomerization. This pore allows the release of proinflammatory cytokines and leads to cell membrane rupture [193]. In this context, the nuclear factor 'kappa-light-chain-enhancer' of activated B-cells (NF-kB), a pivotal pro-inflammatory and pro-apoptotic transcriptional factor, is activated by reactive oxygen species (ROS). NF-kB is responsible for the upregulation of NLRP3 protein, pro-IL-1β, and pro-IL-18, thereby perpetuating a vicious cycle of inflammation and cell death [194, 195]. Moreover, oxidative stress, particularly via lysosomal membrane permeabilization, may trigger pyroptosis in AMD [196, 197]. Drusen components like lipofuscin, C1q, or Aβ-peptide have been reported as possible stimulants for pyroptosis in AMD [198-201]. An interesting study conducted by Wooff and coworkers, investigating the effect of photooxidative stress on retinal degeneration, concluded that caspase 1 related photoreceptor cell death occurs largely independently of NLRP3, thereby suggesting caspase 1 and not NLRP3 as a suitable target in retinal degenerative disorders [191]. A more recent investigation by Sekar et al. on transgenic mice, proposed the pyroptotic pore-protein gasdermin D as a key-player in the escalation of retinal inflammation via the release of IL-1β, therefore indicating this pore-protein a promising therapeutic target in AMD [202].

**Figure 4.**
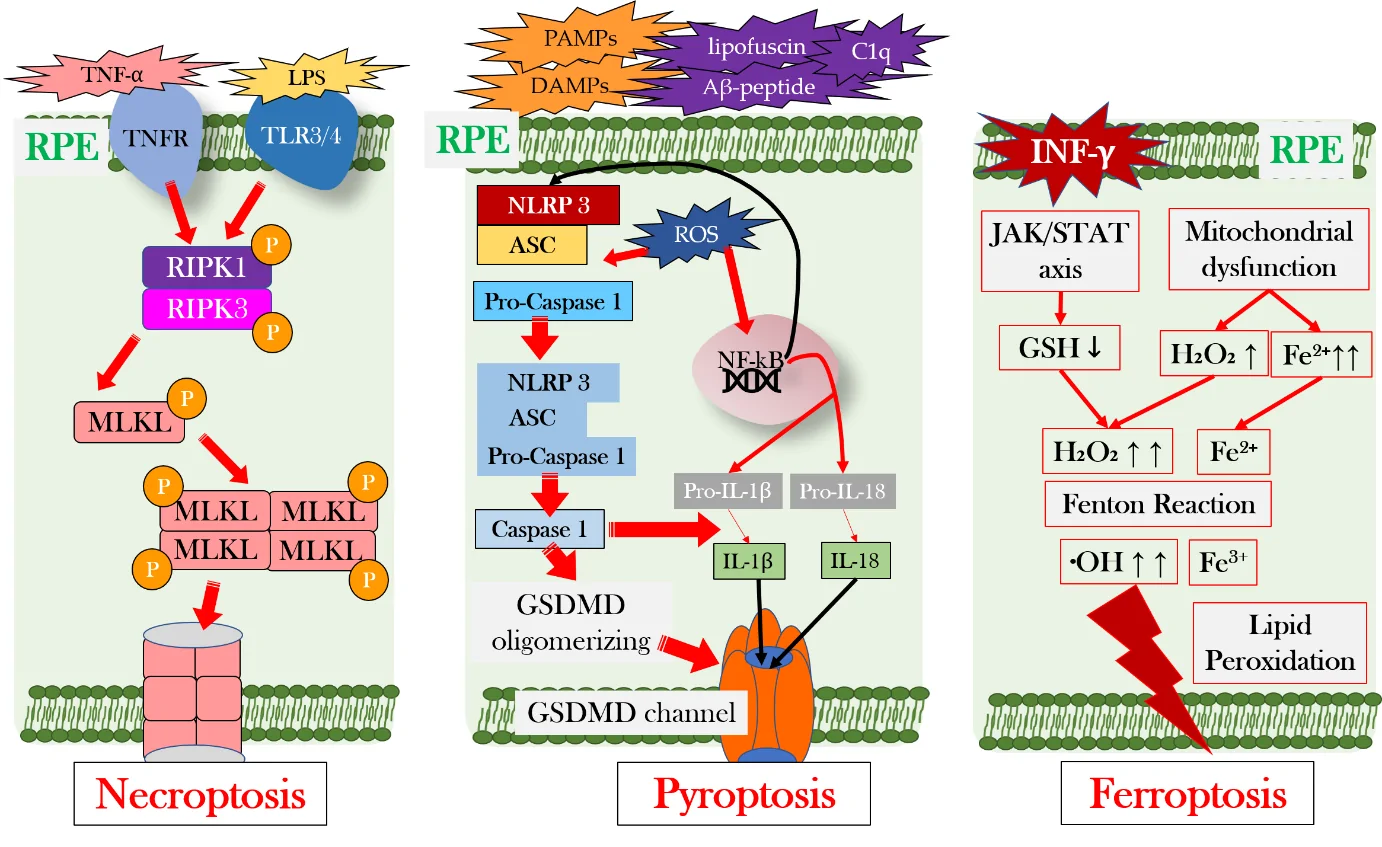
**Overview of the different cell death pathways in AMD-RPE**. RPE: retinal pigment epithelium; IL: interleukin; MLKL: mixed lineage kinase domain-like pseudokinase; RIPK: receptor interacting protein kinase; TNF-α: tumor necrosis factor alpha; TNFR: tumor necrosis alpha receptor; TLR: toll-like receptor; LPS: lipopolysaccharide; DAMP: damage-associated molecular patterns; PAMP: pathogen-associated molecular patterns; NLRP 3: NOD-like receptor, nucleotide-binding domain, leucine-rich-containing family, pyrin domain-containing-3; ASC: adapter protein apoptosis-associated speck-like protein containing a caspase activation and recruitment domain; GSDMD: gasdermin D; NF-kB: nuclear factor 'kappa-light-chain-enhancer' of activated B-cells; ROS: reactive oxygen species; JAK: Janus kinase; STAT: signal transducers and activators of transcription; INF-γ: interferon gamma; GSH: glutathione.

Excess iron has been linked to AMD-RPE, inducing a relatively recently discovered type of programmed cell death known as ferroptosis. AMD is characterized by high lipid peroxidation and iron overload [203, 204]. Several neurodegenerative pathologies, such as Alzheimer’s disease, Parkinson’s disease, Friedreich's ataxia, and Huntington disease have shown evidence of mitochondrial dysfunction accompanied by iron accumulation. It has been speculated that mitochondrial dysfunction, iron excess, and ROS generation may form a positive loop contributing to massive cell death processes in neurodegeneration [205]. Ferroptosis is morphologically observable through specific shrinkage of mitochondria, enhanced membrane density, decreased mitochondrial cristae, and chemically originates via the Fenton reaction, in which H^2^O^2^ and Fe^2+^ react producing Fe^3+^, and importantly, hydroxyl radical (∙OH), causing lipid peroxidation [206]. Iron-overloaded RPE cells develop oxidative stress, eliciting lysosomal membrane permeabilization and ceramide accumulation, while suppressing lysosomal enzymes [207]. Elevated interferon-γ (IFN-γ) in AMD enhances ferroptosis in RPE-AMD via a Janus kinase/signal transducers and activators of transcription (JAK/STAT) molecular pathway [208]. Hypoxia, a condition associated with AMD, may escalate ferroptosis through an increase in the Fenton reaction [209]. Glutathione-specific gamma-glutamylcyclotransferase 1 (Chac1), a mediator of ER stress, has been implicated in ROS-induced ferroptosis in AMD, suggesting a possible treatment target [210]. Figure 4 summarizes the different cell death pathways, possibly responsible for the RPE cell loss in AMD.

### 3.2. Aging, Mitochondria and Inflammation

#### 3.2.1. Aging and Mitochondrial Dysfunction

Intracellular clearance processes cells play a crucial role in maintaining retinal health. Autophagy, a mechanism for intracellular degradation of damaged or unused biomolecules or organelles, is widely employed in eukaryotic cells and is fundamental for cellular homeostasis [211, 212]. RPE cells implement this phenomenon through the formation of extensive double-membrane vacuoles, termed autophagosomes, which sequester undesired and non-functional macromolecules, complexes, or organelles. These are later eliminated by enzymes after fusion with lysosomes [163, 164]. Notably,, RPE physiologically maintains a high rate of autophagy [163]. Dysfunctional autophagy in RPE has been reported to lead to escalated ROS generation, potentially contributing to the initiation of AMD [213, 214].

The functionalities of mitochondria in RPE cells strongly support photoreceptor health, allowing normal vision [215, 216]. Considering possible specific intracellular entities, autophagosomes can specifically target mitochondria in a process known as mitophagy [217]. Various stress stimuli common in the RPE, such as hypoxia, unfolded proteins, and oxidative stress, can induce mitophagy [218, 219]. However, mitophagy tends to decrease with aging [220, 221]. A reduced rate of functional mitophagy can result in reduced clearance of damaged mitochondria, leading to the release of mtDNA fragments and ROS, possibly culminating in AMD pathogenesis [222-224]. Moreover, decreased autophagy flux can lead to the accumulation of lipofuscin, a retinal pigment derived from the phagocytosis of shed POS, in lysosomes [225, 226]. Importantly, lipofuscin is a photoinducible source of ROS and has been described as an initiator of blue light-related damage in RPE cells [227]. Lipofuscin contains vitamin A-derived fluorophores, including A2E, which diminishes mitochondrial energy generation in RPE cells [228, 229]. Moreover, A2-E is a photosensitizing molecule that reduces lysosomal enzymatic function and interferes with cholesterol metabolism [230-232]. The interplay between ROS excess and dysfunctional autophagy plays a pivotal role in the pathophysiology of AMD and has garnered significant scientific interests as one of the more innovative therapeutic strategies to counteract this disorder [233].

In parallel, impaired mitochondrial dynamics in terms of fusion and fission have been reported in senescent cells [234], possibly linked with reduced mitochondrial motility [235]. Importantly, mitophagy and mitochondrial fission activities are interconnected: the process of mitochondrial fission is propaedeutic for the separation of impaired mitochondria from the mitochondrial network before mitophagy [162]. Mitochondrial fission is primarily regulated by the dynamin-related protein 1 (Drp-1) in humans [236]._Mitochondrial dynamics are vital for retaining a balanced level of mitochondrial ROS, buffering the number of dysfunctional units, and avoiding the initiation of a positive loop of ROS and damaged mtDNA [167, 237, 238]. Dysfunction in mitochondrial homeostasis, through abnormal dynamics and metabolic activities, represent a critical sign of advanced cellular age and may evolve into AMD [239]. Triggers related to AMD, such as tobacco smoking and complement activation, may induce mitochondrial fragmentation and dysfunction [240-242]. In addition, a state of hyperfusion, characterized by an abnormal increase of mitochondrial fusion, along with decreased mitochondrial turnover, has been identified as a driver of RPE senescence [162]. Specifically, the PGAM family member 5, mitochondrial serine/threonine protein phosphatase (PGAM5), may be a central player in these events, being indispensable for mitochondrial fission through dephosphorylating of Drp1 [243]. Yu et al. observed in transgenic mice that a PGAM5 deletion causes an aberrant mitochondrial fusion and reduced mitochondrial turnover, leading to elevated cellular ATP and ROS, enhancement of the mammalian target of rapamycin (mTOR) and interferon-regulatory factor (IRF)/ interferon beta (IFN-β) signaling, negative regulation of autophagy, ultimately leading to cellular senescence [244]. Imbalanced mitochondrial fission/fusion may disturb the motility and ATP synthesis in mitochondria, promote ROS generation and mtDNA mutations, and further affect intracellular Ca^2^ concentration, inevitably leading to cell death [162, 245, 246].

Considering that aging is the major risk factor for AMD, it is not surprising that several studies have described age-related impairment of mitochondrial dynamics in this disorder. For example, Bianchi et al. reported a higher number of fragmented mitochondria in RPE cells of individuals affected by GA compared to healthy controls [247]. Mechanistically, an excessive mitochondrial fission, resulting in mitochondrial disintegration or fragmentation, may be a consequence of oxidative stress in neurodegenerative disorders, involving abnormal activation of Drp1, the protein that physiologically serves to eliminate damaged mitochondria during fission [248]. However, a recent study by Fisher and associates, investigating mitochondrial dynamics and mitophagy in RPE from donors with AMD compared to RPE without AMD, assessed that during fission the mitochondrial fission factor (mff), mediator of mitochondrial fission, is higher in AMD-RPE than in controls [249]. In the same study, the authors displayed that lower levels of mitophagy occur in AMD-RPE, indicating a reduced basal level of PTEN-induced kinase 1 (PINK1), a regulator of mitophagy [249]. An in vitro study conducted by Yako and coworkers reported that a suppression of mitochondrial fission using the mitochondrial division inhibitor 1 (Mdivi-1) effectively countered H_2_O_2_-induced damage in an adult retinal pigment epithelial cell line (ARPE-19), an established human RPE cell line. This resulted in preserved cells with reduced release of cytochrome c from the mitochondria and improved mitochondrial function [250]. Another experimental approach to restore the mitochondrial functionality is exogenous mitochondrial transplantation. Noh et al. described that a transplantation of mitochondria isolated from umbilical cord mesenchymal stem cells (UC-MSCs) to senescence-induced dysfunctional mitochondria in ARPE-19 cells attenuates the loss of mitochondrial function, counteracting imbalanced mitophagy and excessive fission [251].

In addition to reduced mitophagy and impaired rates of fission/fusion, a decreased mitochondrial biogenesis has been recorded in the current literature as a characteristic of AMD [167]. Mitochondrial biogenesis is the mechanism through which cells increase mitochondrial mass, enhance mtDNA, its transcription and translation, in an event of mitochondrial self-renewal [252, 253]. Pivotal regulators of biogenesis include suirtin1 (SIRT1), peroxisome proliferator-activated receptor gamma (PPARγ) coactivator-1alpha (PGC-1α), and nuclear factor (erythroid-derived 2)-related factor 2 (Nrf2). PGC-1α, a co-transcriptional factor, activates different agents in the nucleus and mitochondria, promoting mitochondrial biogenesis, degradation by mitophagy and upregulation of antioxidant molecules [254-257]. This transcriptional factor is especially highly expressed in the retina, where it regulates the antioxidant capacity of RPE cells, and also pathological neoangiogenesis in CNV [258-260]. PGC-1α can be triggered by two central agents: the AMP-activated protein kinase (AMPK) and the NAD^+^-dependent deacetylase, SIRT1 [261]. In fact, SIRT1 and AMPK interact modulating autophagy and mitophagy, further supporting the mitochondrial biogenesis by activating PGC-1α [262]. Nrf2 critically interacts with the co-factor PGC-1α, operating in the mitochondrial biogenesis perpetuating a regulatory loop, as suggested by Gureev et al [263]. Moreover, poly(ADP-ribose) polymerase-2 (PARP-2) is a negative regulator of SIRT1, and its deletion in transgenic mice has been described to induce upregulation of SIRT1 and PGC-1α, enhancing mitochondrial biogenesis [264]. Furthermore, the mTOR signaling pathway controls cell growth and has the ability to suppress autophagy [265]. An interesting investigation by Zhang and colleagues examined the dysregulated molecular pathways in AMD on isolated native RPE cells from normal and AMD deceased donor eyes and found upregulated PARP-2, reduced NAD^+^, dysregulated AMPK/SIRT1/PGC-1α axis, and overactive mTOR in AMD-RPE [266].

**Figure 5.**
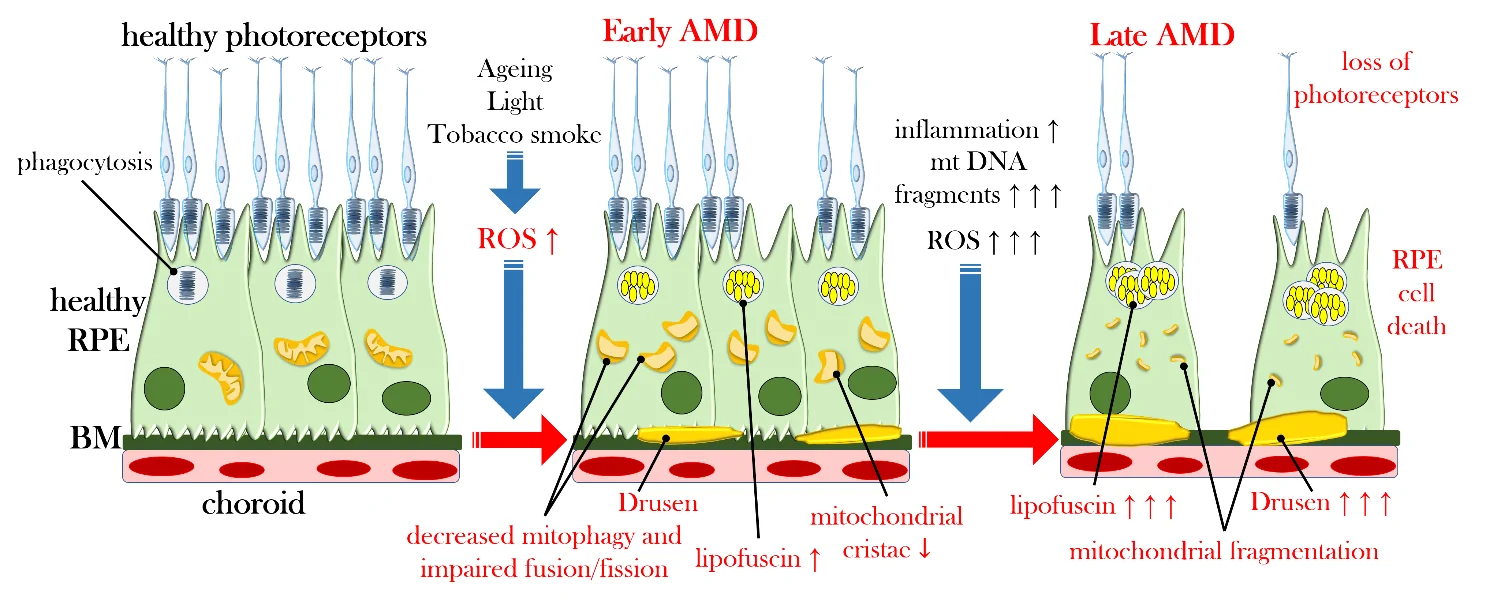
Triggers of structural changes in the RPE and morphological characteristics of healthy and AMD-RPE. RPE: retinal pigment epithelium; AMD: age-related macular degeneration; BM: Bruch’s membrane; mtDNA: mitochondrial DNA.

Taken together, from a cellular point of view, AMD-RPE cells display a reduced number of mitochondria, with loss of cristae, along with a defective fusion/fission ratio and accumulation of lipofuscin [162]. Figure 5 provides a schematic representation of the main triggers of structural cellular changes and of morphological intracellular characteristics of AMD-RPE.

#### 3.2.2. The Concept of Inflamm-Aging and its Relevance in AMD

Advanced age is invariably accompanied by a decline in the immune system [267], which has been described as an immunoremodeling event, possibly arising from chronic insults over time [268]. Cellular senescence, considered an adaptation to noxious stimuli in both mitotic and postmitotic cells, is triggered by tumor suppressor proteins, leading to the establishment of an inflammatory secretome [269]. T cells in this context tend to shift towards an innate-like functionality as terminal differentiated cells, progressively losing their relevant antigen specific activity [270]. Immunosenescent cells exhibit distinct morphological and functional characteristics, including an accumulation of DNA damage foci like double-strand breaks, impaired mitochondrial metabolism, heightened activity of senescence-associated β-galactosidase (SA-β-Gal), and increased glycolytic rates [269]. These cells gradually lose their immune cell effector activity, transitioning into senescence-associated secretory phenotype (SASP), where they contribute to the overproduction of pro-inflammatory mediators [271]. There is growing scientific interest in the literature regarding the link between SASP and AMD, with potential therapeutic implications through senolytic or senostatic drugs targeting senescent cells to combat AMD [272, 273]. Senescent RPE cells exhibit cellular markers of SASP, such as p16^INK4A^, p21^CIP1^, and SA-β-Gal [274], and express pro-inflammatory mediators like IL-8, IL-33, MMP9 and VEGF [275-277]. Supporting this association, serum and aqueous humor of AMD patients contain a myriad of proinflammatory cytokines, including TNF-α, IL-1β, IL-17, IL-6, and IL-8 [278, 279].

Immunosenescence intricately connected with the concept of inflamm-aging, although they differ in certain aspects [268]. In a context of inflamm-aging, the immunoremodeling and adaptive processes of immunosenescence, as well as anti-inflamm-aging compensatory mechanisms, tend to decrease, failing to balance the chronic inflammatory state of the retina, known as “para-inflammation”, and leading to an escalation of inflammation [268]. Consequently, an aberrant chronic inflammation state emerges, accompanied by a dramatic reduction in the physiological immunosuppressive activity of the retinal environment. In the retina, RPE cells play a crucial role in immunoregulation and immunosuppression [280, 281]. However, during aging, these immunomodulatory mechanisms tend to deteriorate, accompanied by abnormal proinflammatory cytokine production, innate and adaptive immune activation, and complement activation [282-284]. Notably, retinal microglia, involved in physiological immunomodulation and expressing basal levels of MHC class II like RPE cells [285], exhibit increased expression of MHC II during aging [286]. Senescent microglial cells have been reported to contribute to AMD by amplifying chronic inflammation, altering expression of NF-kB, CFH, and CFB under specific stimulation, such as A2E accumulation, as well as impacting the activity of RPE cells [287-289]. A recent study on a murine model by Boyce et al. reported that RPE-related mediators activate microglial cells via an Akt2 pathway, leading to the upregulation of adhesion factors for neutrophils [290]. The authors also demonstrated that targeting Akt2 signaling suppressed proinflammatory microglial activation and neutrophil recruitment [290]. Collectively, RPE cells and retinal microglia engage in a positive loop where microglia promote changes in RPE structure and gene expression. In turn, RPE triggers a more chemoattractive framework for the retention of additional microglia, collectively contributing to immune privilege compromise in the outer retina and perpetuating a state of chronic neuroinflammation, pivotal in AMD [291, 292].

### 3.3. Complement System, Altered Redox Status, and Autoantibodies

#### 3.3.1. Complement System and its Role in the Pathogenesis of AMD

The complement system is a protein network comprising of approximately fifty components or complement factors and plays a fundamental role in detecting and regulating the elimination of pathogens and debris, such as dead cells [293]. Complement factors can switch from inactive to active forms through proteolytic processes activated by one of the following three cascades [294-296]:

1. Classical pathway: Activated by an antibody-dependent binding of complement factor 1q (C1q) to the pathogen surfaces.
2. Alternative pathway: Physiologically active at basal levels due to spontaneous hydrolysis of C3 to C3(H_2_O) in a process known as complement ‘tick-over’.
3. Lectin pathway: Triggered by the recognition of pathogen surfaces or apoptotic/necrotic cells, mediated by a pattern-recognition molecule called mannose-binding lectin (MBL).

These cascades aim to form the C3 convertase protein complex, cleaving C3 into C3a and C3b. C3b, a crucial component, facilitates the amplification loop of complement activation by generating the C5 convertase, leading to the cleavage of C5 into C5a and C5b. C5b, in turn, initiates the formation of the membrane attack complex (MAC or terminal complement complex, TCC), causing pore formation and cell lysis. Specific polymorphisms of CFI and CFH, along with C2, C3, CFB, and C9, have been associated with AMD [73-77]. CFI and its cofactor CFH act as negative regulators, suppressing C3b into its inactive form (iC3b) [297].

Overactivation of the complement pathways is a pathogenetic driver in several disorders, and its association with the occurrence and progression of AMD has been extensively studied in the last decades [298]. Numerous studies have demonstrated numerous studies demonstrating elevated plasma levels of complement components in AMD patients, including C3a, CFI, C4a, C5a, and MAC [299-303]. Remarkably, complement activation levels correlate with disease stage, with higher levels observed in intermediate and late dry AMD compared to early stages [304]. Notably, complement inhibition has proven effective in both dry and wet AMD models [304, 305].

Activated complement can induce cell lysis and death, excess phagocytosis, inflammation, and an amplification loop, perpetuating chronic inflammation and upregulating NF-kB in AMD [294, 306]. Anaphylatoxins C3a and C5a recruit immune cells, contributing to chronic inflammation, and C1q has been reported to activate the NLRP3 inflammasome [198, 307]. Dysregulation of complement functionality is observed not only among immune cells but also in resident microglia, contributing to RPE degeneration [308].

Anomalous activation of the alternative pathway is particularly relevant in AMD pathophysiology [297, 298]. In support of this hypothesis, gene associations with some variants of CFH, CFB, CFI, C2, C3, C9, elevated levels of MAC in the choriocapillaris, and presence of complement factors in drusen have been reported [73-77, 309, 310].

Downregulation of membrane-associated regulators, such as membrane cofactor protein (MCP) and decay-accelerating factor (DAF), in AMD results in dysregulated CFH and CFI activity, leading to aberrant activation of the alternative pathway [311-314]. This increased activation contributes to MAC excess and deposition into Bruch's membrane (BM) and choriocapillaris, disrupting the tight interaction between RPE-BM-choroid [310].

#### 3.3.2. Reactive Oxygen Species Excess and Complement Anomalies

An excess of ROS can adversely affect complement functionality in various ways [315]. Chen et al. demonstrated in cultured RPE cells from mouse or human donors that the phagocytosis of oxidized POS by RPE cells downregulates the synthesis of CFH in RPE [316]. Cigarette smoke, a well-known trigger of oxidative stress, has been reported to activate C3 and the alternative complement pathway in an *in vitro* investigation [317]. Wang et al. demonstrated that APRE-19 cells, in response to cigarette smoke-induced oxidative stress, overexpress C3, CFB, and C5, while downregulating negative regulators of the complement system such as CD46, CD55 and CD59 [311]. Dysregulated expression of CD46 in RPE cells has been suggested as one of the earliest modifications occurring in GA [318], and its decreased expression has been linked with a drusen-type disorder in a murine model [319].

Marazita et al. demonstrated on ARPE-19 cells that the cigarette smoke-induced excess of H_2_O_2_ leads to a transition to a senescent phenotype in RPE cells. This transition is marked by an upregulation of senescence markers such as SA-β-Gal and p16^INK4a^ and p21^Waf-Cip1^, an overexpression of VEGF, IL-6, and IL-8, and a downregulation of CFH [320]. Thurman et al. found in ARPE-19 cells that treatment with H_2_O_2_ enhances the cell surface deposition of C3 while decreasing the surface expression of complement inhibitors such as CD55, CD59, and DAF [321]. Armento and associates demonstrated that a loss of CFH in human RPE cells induces complement dysregulation, involving upregulation of C3 and CFB, downregulation of C5, and an overexpression of proinflammatory cytokines such as IL-6 and IL-8. This suggests that CFH and NF-kB regulatory activities in complement and inflammation, respectively, may act synergistically to maintain RPE homeostasis. In cases of dysregulation in one of these modulations, RPE may transition into an AMD-like phenotype [322]. Conversely, a lack of C3 in a transgenic murine model showed improved retinal thickness, higher antioxidant levels, and lower pro-oxidative markers [323]. A recent study by Gurubaran et al. on transgenic mice lacking Nrf2 and PGC-1α, which develop oxidative stress, reported enhanced C5a levels not dependent on C3, possibly related to CFH and its co-factor thrombin [324]. A prior study by Mulfaul et al. suggested a possible role of Toll-like receptor 2 (TLR2) as a bridge between oxidative stress and complement activation in retinal degeneration. TLR2 was found to favor the expression of C3 and CFB, the pivotal factors of the alternative pathway, in both RPE cells and mononuclear phagocytes. Lack of TLR2 resulted in decreased C3 fragments in the outer retina, preserving photoreceptors from oxidative stress-induced degeneration [325].

CFH appears to play a crucial role in oxidative stress-mediated damage to RPE cells by allowing aberrant activation of the alternative complement pathway. A murine model lacking CFH demonstrated a link between CFH and mitochondrial dysfunction, observing abnormal size and reduced mtDNA, along with reduced ATP generation [326]. In a cybrid model composed of mitochondria obtained from patients with AMD and ARPE-19 cells without mitochondria, an increase in complement factor activators, such as CFB, and a decrease in complement factor inhibitors, such as CFH and CFI, were observed [327]. Beyond mitochondria, complement activation may dramatically impact the autophagic flux and lysosomal function in RPE cells. Depletion of C3 in a murine model resulted in improved autophagy in RPE [323]. The Y402H polymorphism of CFH, a major risk factor for AMD, has been associated with pathological lipid accumulation in RPE cells. iPSC-derived RPE cells harboring the Y402H variant displayed increased accumulation of lipid droplets and drusen-like deposits

A major risk factor for AMD is the Y402H polymorphism of CFH. Borras et al. showed that treatment with an oxidized lipid, 4-hydroxy-2-nonenal, produced by photoreceptors, both ARPE-19 and iPSC-derived RPE cells were preserved from oxidative stress-mediated injuries in the presence of the full-length human recombinant CFH, whereas the variant Y402H did not show such a fundamental cytoprotective function [328]. Moreover, in iPSC-derived RPE cells harboring the Y402H variant, an augmented deposition of C5b9 on malfunctional lysosomes could be observed [329].

Interestingly, the CFH Y402H variant has also been linked with increased lipid accumulation in RPE cells. Of note, lipids are major components of drusen. For instance, in iPSC-derived RPE cells carrying the Y402H variant, displayed increased accumulation of lipid droplets and drusen-like deposits [330]. An investigation by Acar et al. on the blood of individuals with AMD revealed an association between complement activation and large or extra-large HDL [331]. Intriguingly, complement factors including CFH, have been identified as components of large HDL [332], suggesting that CFH may be sequestered by HDL, inhibiting complement activation, or that CFH may bind to lipoproteins to block the inflammatory response initiated by oxidative LDL (oxLDLs) [298, 333]. CFH is in fact indeed capable of reducing the uptake of oxLDLs in ARPE-19 cells [334]. Importantly, the Y402H variant disrupts lipid metabolism in RPE cells due to the disability of CFH to bind oxidative byproducts, such as OxLDLs and MDA [335-337].

#### 3.3.3. Oxidative Processes, Autoantibodies and Complement Activation

High levels of retinal autoantibodies have been intriguingly detected in patients with both dry and wet AMD, in comparison to healthy controls. These levels correlate with the disease stage and demonstrate binding with antigens playing various roles in inflammation, autophagy, protection from oxidative stress, and apoptosis [338-342]. For example, the level of autoantibodies against an inflammatory protein like transferrin has been shown to be downregulated in individuals with early AMD and soft drusen [342].

The genesis of retinal autoantibodies in AMD could be attributed to processes of lipid peroxidation in the context of ROS overabundance, stemming from events like photo-oxidation. These processes lead to the formation of novel antigens on the cellular surface that immune cells do not recognize as self-antigens [343]. In the retina, the oxidation of docosahexaenoate (DHA)-containing lipids results in the formation of carboxyethylpyrrole (CEP) protein derivatives [344]. Elevated levels of CEP proteins have been observed in drusen and blood of AMD patients compared to healthy controls [108, 345]. Notably, CEP has been identified as an oxidized component of the pigment lipofuscin, along with molecules like A2E [346]. These oxidized molecules play a central role in triggering the production of autoantibodies, likely through an interaction between autoreactive T and B cells, ultimately leading to chronic inflammation and cell apoptosis [343, 347, 348].

The activation of the classical pathway through autoantibodies activating C1q has been reported in mice. In these cases, the formation of antibodies against oxidative byproducts in the outer retina can drive to classical complement activation, subsequently leading to C3 deposition in BM and macrophage infiltration in RPE cells [338, 345, 349-351]. Moreover, ROS-related activation of C1q triggers the NLRP3 inflammasome in cultured RPE, resulting in cell death [198].

A noteworthy investigation on ARPE-19 cells highlights the connection between oxidized lipids, autoantibodies and complement activation [352].

**Figure 6.**
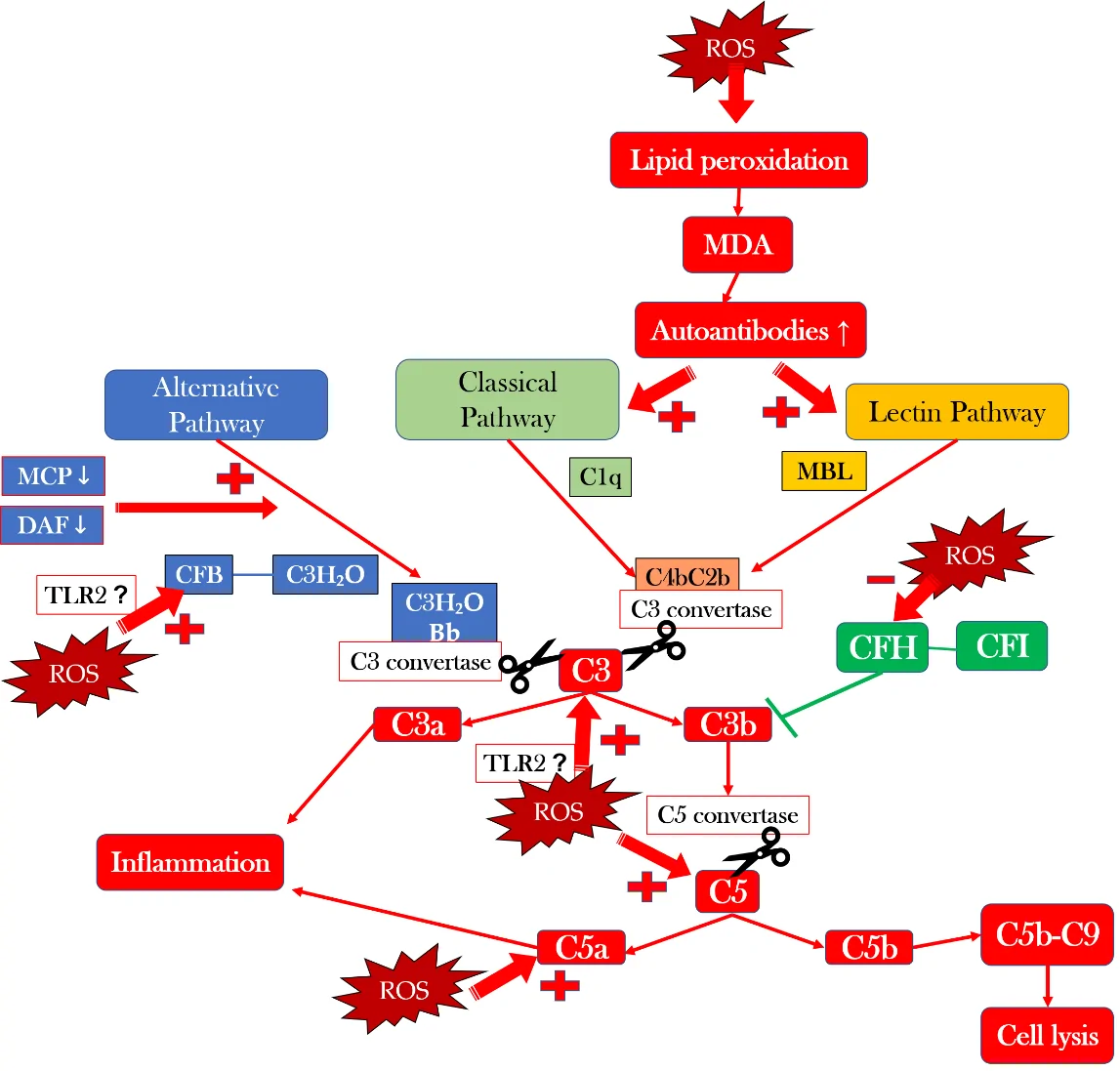
**Interrelation between oxidative stress, complement activation and generation of autoantibodies in AMD**. CF: complement factor; MBL: mannose-binding lectin; MCP: membrane cofactor protein; DAF: decay-accelerating factor; TLR: toll-like receptor; ROS: reactive oxygen species; MDA: malonyldialdehyde.

The study specifically reveals that an excess of ROS promotes the formation of neoepitopes on RPE cell surfaces containing phospholipids, such as MDA. Autoantibodies present in normal serum can recognize these oxidative stress-induced surface modifications, subsequently activating the complement cascade via the lectin pathway [352]. Figure 6 presents the intricate interrelation between oxidative stress, complement pathway anomalies and autoantibodies in AMD.

## 4. Novel Curative Strategies

### 4.1. Complement Inhibitors

#### 4.1.1. Pegcetacoplan: A C3 Inhibitor

Pegcetacoplan is a compound constituted by two analogous pentadecapeptides connected by a linear polyethylene glycol that enhances the molecule’s half-life [353]. This molecular entity has the ability to bind to both C3 and C3b, thereby suppressing their activities and leading to the inhibition of an overactivated complement system [354].

One of the pivotal clinical trials investigating the therapeutic potential of pegcetacoplan in GA is the phase 2 FILLY study (NCT02503332). This study involved 246 GA patients treated for 12 months, with follow-up assessments at 15 and 18 months post-therapy initiation, utilizing fundus autofluorescence imaging to detect atrophy extension [355]. Significantly, the FILLY trial demonstrated that local C3 inhibition with pegcetacoplan resulted in a statistically significant reduction in GA growth compared to sham treatment [355]. A post hoc analysis conducted by Riedl and colleagues, utilizing spectral-domain optical coherence tomography (SD-OCT), further revealed that pegcetacoplan could also mitigate photoreceptor thinning and loss [356]. Interestingly, according to another post hoc analysis, SD-OCT emerged as a more sensitive monitoring tool during GA therapy compared to fundus autofluorescence [357]. This research group went on to showcase the benefits of pegcetacoplan therapy in evaluating disease activity and progression using SD-OCT supported by automated deep learning assessment—an automated artificial intelligence-based tool [358]. The findings confirmed that pegcetacoplan significantly decelerates GA lesion progression rates compared to sham, with a slower growth rate observed toward the fovea [358].

Recently, Heier and associates reported on two fundamental multicenter, randomized, double-masked, sham-controlled phase 3 clinical trials on pegcetacoplan—OAKS (NCT03525613) and DERBY (NCT03525600). These trials included 637 and 621 patients, respectively, and both confirmed the efficacy of pegcetacoplan, utilizing criteria similar to those in the FILLY study, in slowing the growth rate of GA lesions, maintaining an acceptable safety profile [359].

Notably, in both the FILLY and OAKS/DERBY trials, pegcetacoplan did not exhibit any benefits on visual acuity, and a progressive visual deterioration was observed across all study groups [355, 359]. Additionally, pegcetacoplan presented significant adverse events, particularly a surprisingly elevated rate of progression from GA to choroidal neovascularization (CNV). In the FILLY trial, this rate reached 20.9% in the monthly treated subgroup, 8.9% in the two-monthly treated subgroup, and 1% in the sham-treated eyes [355]. Occurrences of CNV were also noted in 11%, 8%, and 2% of patients in the OAKS trial, and in 13%, 6%, and 4% of patients in the DERBY trial, corresponding to those treated monthly, two-monthly, and sham-treated, respectively, at the 24-month mark [359]. Another observable side effect in the FILLY trial was the occurrence of endophthalmitis following injection, with 2.3% of eyes in the monthly-injected subgroup experiencing this serious ocular infection [355], while the rates of endophthalmitis were lower in the DERBY and OAKS trials [360].

#### 4.1.2. Avacincaptad pegol: A C5 Inhibitor

Avacincaptad pegol, a pegylated RNA aptamer with the ability to bind and inactivate C5 [361], underwent evaluation for its efficacy and safety in patients with geographic atrophy (GA) through the randomized, double-masked, and sham-controlled phase 2/3 GATHER1 clinical trial (NCT02686658) [361]. The trial comprised two parts: the first involved 77 patients randomized into three groups receiving 1 mg of avacincaptad, 2 mg of avacincaptad, or sham, via monthly intravitreal injections. In the second part, 209 patients were randomized into groups receiving 2 mg of avacincaptad, 4 mg of avacincaptad, or sham, also via monthly intravitreal injections [361]. GATHER1 demonstrated a reduction in the growth rate of GA lesions, with avacincaptad pegol at both 2 mg (27%) and 4 mg (28%) showing efficacy compared to the sham group at month 12 [361]. An 18-month follow-up revealed a further reduction in GA lesion size of 28.1% and 30% in the 2 mg and 4 mg arms, respectively, compared to the sham group [362]. Notably, similar to pegcetacoplan, avacincaptad pegol in GATHER1 exhibited an evolution into choroidal neovascularization (CNV) in 9%, 9.6%, and 2.7% of patients in the 2 mg, 4 mg, and sham subgroups, respectively [361].

Recently, Khanani et al. published the 12-month results of the confirmatory randomized, double-masked, sham-controlled GATHER2 clinical trial (NCT0443 5366), a 24-month phase 3 study involving 448 patients randomized to receive 2 mg of avacincaptad pegol or sham injections [363]. At month 12, the avacincaptad pegol subgroup showed a significant decrease in GA growth area compared to the sham group, with a 14% difference between the two subgroups in total atrophic areas [363]. However, at month 12, exudative macular neovascularization was observed in 5% of patients receiving avacincaptad pegol 2 mg and in 3% of patients in the sham group, with no cases of intraocular inflammation or endophthalmitis recorded [363].

#### 4.1.3. Other Complement Inhibitors

Lampalizumab, an antigen-binding fragment of a humanized monoclonal antibody, binds and inactivates CFD [364], a serine protease crucial for the formation of the C3 convertase in the complement alternative pathway [365]. The multicenter, randomized, sham-controlled MAHALO phase 2 clinical trial (NCT01229215) explored the impact of lampalizumab on GA patients. Participants received monthly intravitreal injections (42 patients) or injections every other month (41 patients) versus a sham control group (40 individuals) for 18 months. The monthly subgroup showed a 20% decrease in GA growth compared to the sham treatment [366]. However, the confirmatory phase 3 clinical trials, CHROMA (NCT02247479) and SPECTRI (NCT02247531), involving 906 patients, demonstrated that lampalizumab did not reduce GA enlargement compared to sham-controls over 48 weeks of treatment, with different dosing regimens [367].

Along with avacincaptad pegol, also eculizumab and tesidolumab were designed as C5 inhibitors and tested for the management of GA in dedicated clinical trials. Eculizumab, a murine humanized monoclonal antibody inhibiting C5 [360], underwent evaluation in the COMPLETE study, a prospective, double-masked, randomized phase 2 clinical trial with 30 GA patients. Unfortunately, it reported a lack of effectiveness in reducing GA growth [368]. Tesidolumab, a humanized monoclonal antibody against C5, similarly did not show efficacy in decreasing GA growth after 12 months in another clinical trial (NCT01527500) [364].

In summary, while lampalizumab, eculizumab, and tesidolumab did not demonstrate benefits in decreasing the GA growth rate, trials on pegcetacoplan and avacincaptad pegol have proven the effectiveness of these molecules in reducing GA lesion growth compared with sham-controls. These drugs have been collectively well tolerated, forming the basis for their FDA approvals. However, they also exhibited notable adverse events, particularly a dose-dependent increase in the rate of evolution into CNV in treated eyes [369]. This underscores the importance of strict patient selection [17, 370] and caution in drug administration, emphasizing the need for rigorous monitoring in clinical practice [371].

### 4.2. Experimental Targeting Options

#### 4.2.1. Nrf2, SIRT1, and PGC-1α as Molecular Targets

In a murine model treated with NaIO_3_, Hsu and colleagues demonstrated that quercetin, a polyphenolic flavonoid compound with antioxidative properties found in fruits, vegetables, and various berries, can counteract the overabundance of ROS by modulating mitochondrial ROS homeostasis. This modulation occurs through the acetylation of SOD2 via the Nrf2/PGC-1α/Sirt1 signaling pathway [372]. Supporting these findings, a prior study by Shao and co-workers revealed that quercetin enhances the expression of Nrf2 target genes, such as HO-1 and HQO-1, in retinal tissues of a GA murine model. However, this effect was not observed in Nrf2 knockout mice, thereby confirming the mechanistic pathway of quercetin in mitigating AMD [373].

Another noteworthy flavonoid compound is chrysoeriol, found in plants of the species *Perilla frutescens*. Kim and colleagues demonstrated that chrysoeriol protects ARPE-19 cells from oxidative stress and mitochondrial dysfunction induced by H_2_O_2_. This protection is achieved through an increase in OPA1 and a decrease in DRP1, thereby balancing mitochondrial dynamics and activating the antioxidant response through Nrf2 [374].

Sulforaphane, a natural isothiocyanate present in cruciferous vegetables, exhibits antioxidative effects on particulate matter-induced oxidative stress in ARPE-19 cells [375]. Additionally, it demonstrates anti-inflammatory effects on lipopolysaccharide (LPS)-induced inflammatory injury in ARPE-19 cells by blocking NF-kB activation [376]. Furthermore, Wang and Tang found that sulforaphane ameliorates cell viability and reduces apoptosis in amyloid beta (Aβ)-induced inflammatory injured ARPE-19 cells by modulating the PARP1/SIRT1 pathway [377].

Dimethyl fumarate (DMF), a methyl ester of fumaric acid found in nature in *Fumaria officinalis* and approved for psoriasis and multiple sclerosis, has been extensively reviewed by Manai et al. for its drug potential [378]. Mechanistically, DMF acts as a Nrf2 activator by binding and inhibiting Keap1, the cytoplasmic repressor of Nrf2. This action allows the translocation of Nrf2 into the nucleus and the upregulation of antioxidant genes such as HO-1 and HQO1. In the context of AMD, Catanzaro and associates conducted an *in vitro* study on wild-type and Nrf2-silenced ARPE-19 cells exposed to various pro-oxidant AMD-related stimuli. Their findings suggested that DMF did not possess direct antioxidant properties and lacked protective effects in Nrf2-silenced cells upon injury [379]. Shu and colleagues also reported on the anti-inflammatory features of DMF in mature human primary RPE treated with TNF-α, demonstrating that a pre-treatment with DMF blocked TNFα-induced inflammatory activation of RPE, reduced levels of pro-inflammatory markers, and prevented TNFα-induced mitochondrial dysfunction and morphological anomalies [380]. Building on this knowledge, the LADIGAGA phase 2 study (NCT04292080), a dedicated randomized and open-labeled clinical trial, commenced in 2022 with the aim of testing the effectiveness of DMF in 30 patients with GA. The primary completion date of the trial is estimated for February 2024.

The carotenoids lutein and zeaxanthin also target the Nrf2 molecule, and several preclinical studies have demonstrated their efficacy in combating oxidative stress in the retina [30, 381-385], alongside possessing notable anti-inflammatory activity [386]. The randomized controlled trial AREDS2 assessed the suitability of lutein/zeaxanthin as a replacement for β-carotene in supplementary diets, aiming to slow the progression of late-stage AMD [387]. A recent systematic review by Evans and Lawrenson cautiously affirmed these findings, highlighting the antioxidant benefits of lutein/zeaxanthin in late AMD [388].

Carnosine, a naturally occurring endogenous dipeptide recognized for its antioxidant properties [389], has the ability to activate Nrf2 [390]. Decreased levels of carnosine have been observed in patients with AMD, prompting investigations into its effectiveness in combating oxidative stress in AMD. In ARPE-19 cells treated with Aβ oligomers—an in vitro model of AMD—Caruso et al. demonstrated that carnosine can counteract ROS excess, inflammation, and the destruction of ZO-1 tight junction protein induced by Aβ oligomers, resulting in a neuroprotective effect [391].

Alpha-lipoic acid (ALA), a naturally occurring organosulfur synthesized by plants and detectable as an endogenous molecule in humans [392], exhibits antioxidative properties by scavenging ROS and regenerating glutathione. It also indirectly functions as an antioxidant by activating Nrf2 [393, 394]. Building on this knowledge, Tao et al. determined in a randomized clinical trial involving 100 patients with dry AMD that ALA treatment induces a statistically significant increase in serum SOD activity and an improvement in low vision quality of life, suggesting ALA as potential drug in AMD [395]. However, a more recent dedicated phase 2 clinical trial reported no benefits of ALA treatment on patients with GA in terms of decreasing atrophic areas and enhancing visual acuity [396].

Cho and coworkers recently demonstrated on blue-light induced damaged ARPE-19 cells and murine retinal cells that *Panax ginseng* berry extracts can antagonize cell injuries, through the activation of the SIRT1/PGC-1α axis and attenuation of NF-kB. This collective inhibition of inflammation and apoptosis proves beneficial [397]. Tang and colleagues showed that treatment of ARPE-19 cells with arbutin, a plant-derived glycosylated hydroquinone extracted from the bearberry *Vaccinium vitis-idaea L*, attenuated ROS-related apoptosis and senescence by activating SIRT1. This activation was evidenced by the increase in downstream FoxO3a and PGC-1α/β, related to mitochondrial biogenesis, and suppression of NF-κB. These findings were further validated in a murine model treated with sodium iodate (NaIO_3_), a well-known chemical oxidant capable of damaging RPE cells [398]. Wan et al. assessed both *in vitro* and *in vivo* AMD models, demonstrating that grape seed proanthocyanidin extract mitigated RPE senescence via SIRT1 activation and NLRP3 suppression [399]. Numerous other preclinical investigations tested the use of naturally occurring molecules on ARPE-19 cells exposed to oxidative stress, determining benefits through activation of the Nrf2 signaling, for example: aloperine [400], madecassoside [401], celastrol [402], phloretin [403], luteolin [404], extracts of *Tribulus terrestri* [405], and phillyrin [406]. Intriguingly, in recent years, some synthetic molecules have been designed to replicate the characteristics of Nrf2 activators. One such molecule is RS9, a triterpenoid obtained from microbial transformation products [407], which has been assessed for its ability to mitigate CNV lesions in a monkey model [408]. Saito and associates showed that RS9 can promote the phagocytosis of POS in RPE cells in a p62-independent manner, modulating not only Nrf2 but also the activation of AMPK [409]. Similarly, another synthetic triterpenoid, RTA408, has been tested for its property to activate Nrf2, showing to preserve primary human RPE cells from oxidative stress [410].

#### 4.2.2. Autophagy Enhancers

Enhanced autophagy has been associated with protective effects in response to oxidative stress in RPE cells [162]. Consequently, both experimental molecules and established medications capable of activating autophagy have been explored as potential drugs in AMD.

For example, Jin and colleagues demonstrated that curcumin, a polyphenol extracted from the *Curcuma longa* plant (turmeric), triggers autophagy and mitigates oxidative stress and apoptosis in human umbilical vein endothelial cells damaged by high glucose exposure [411]. In ARPE-19 cells, Lin et al. later confirmed these findings, demonstrating that a curcumin metabolite, specifically hexahydrocurcumin, enhances autophagic flux, reduces oxidative stress and endoplasmic reticulum stress, ultimately reversing blue light-induced cell death [412]. Munia and associates revealed that lutein and curcumin can prevent a blockage of autophagic flux in ARPE-19 cells, preserving them from ROS-induced cell death [413].

Resveratrol (RSV), a phytoalexin found in various plants and vines with significant antioxidative features, has been extensively studied for its multiple mechanisms of action. Several studies demonstrated that this compound activates the SIRT1/PGC-1α axis, hereby guiding to an enhanced mitochondrial biogenesis and functionality [414-418]. RSV has also been studied in various AMD models. For example, Josifovska et al. demonstrated in ARPE-19 cells an anti-inflammatory and anti-apoptotic effect of RSV, along with enhanced autophagic induction, as indicated by an increased microtubule-associated protein 1 light chain 3 (LC3) II/I ratio, a marker of autophagy, and a decreased p62 expression [419]. Yang et al. reported an antiapoptotic effect of RSV in RPE cells exposed to ROS, regulating SOD activity and activating Bcl-2 expression [420]. Neal et al. found that RSV can preserve cultured human RPE cells from oxidative damage induced by hydroquinone, not only by retaining cell viability, improving mitochondrial bioenergetics, and upregulating antioxidant agents such as HO-1, but also by counteracting endoplasmic reticulum stress and downregulating CHOP [421]. Bhattarai et al. observed antioxidant activity and improved cell viability in ARPE-19 cells treated with RSV, along with an anti-inflammatory effect through a decrease in IL-8 and MCP-1 [422]. Courtaut and coworkers demonstrated in a murine model of CNV that a supplementation with Resvega®, a nutraceutical formulation of omega-3 fatty acids and RSV, significantly reduced CNV, highlighting an anti-angiogenetic effect of RSV [423]. This group further showed a prolonged effect of bevacizumab in undifferentiated ARPE-19 cells through supplementation with Resvega®, suppressing VEGF-A secretion by attenuating both the PI3K/Akt/mTOR and NFκB signaling pathways [424]. Notably, Bhatt and associates developed a nanodelivery system for RSV-loaded poly-DL-lactide-co-glycolide (PLGA) nanoparticles and tested it in ARPE-19 cells, concluding its suitability to enhance the cellular delivery of RSV and augment its anti-angiogenetic effect by inhibiting VEGF expression [425].

Hyttinen and colleagues reviewed the potential of metformin and RSV to modulate the activity of PGC-1α, leading to improved mitochondrial function, autophagic processes, and antioxidant response in AMD [426]. Metformin, a widely used medication for type 2 diabetes, has been reported to trigger autophagy in RPE cells. Zhao et al. described a protective effect of metformin against ROS-related damages in RPE cells through augmented autophagy by activating the AMPK pathway [427]. Mechanistically, metformin may indirectly trigger AMPK by regulating the AMP/ATP ratio through the blockade of complex I of the electron transport chain [428]. In addition, metformin supports mitochondrial biogenesis, likely via PGC-1α activation [429]. It is not surprising that the use of metformin in the diabetic population has been associated with decreased odds of developing AMD [430-432]. Intriguingly, a retrospective study by Eton et al. [433] and a dedicated clinical trial (NCT02684578) did not show a significant impact of metformin on the progression of GA [434].

#### 4.2.3. Zinc

RPE cells exhibit relatively high concentrations of zinc, approximately 300 µg/g of dry tissue [435]. A deficiency in zinc is commonly associated with malnutrition, particularly in the elderly, which suggests the hypothesis that a low zinc diet may play a role in the pathophysiology of AMD. Existing literature reports a reduced content of zinc in human RPE affected by AMD [436-439]. A recent *in vitro* study by Álvarez-Barrios and colleagues identified altered zinc homeostasis in a model of early AMD, with a significant intracellular decrease in zinc concentration and a massive downregulation of zinc-regulating proteins, known as metallothioneins [440]. Zinc exhibits anti-inflammatory features and interacts with the complement system, implying its potential role in modulating complement-related inflammation in AMD [441-443]. Additionally, zinc possesses antioxidant properties by upregulating Nrf2, serving as a co-factor of SOD, and inhibiting NOX [444, 445]. Lu et al. demonstrated that zinc is a substantial factor for the activation of the PGC-1α/Nrf2 axis in human primary endometrial stromal cells [446]. Zinc has also been reported to modulate autophagic fluxes [225]. Interestingly, in AMD, alongside lipofuscin accumulation, there is a reduction in melanosomes, the principal storages of zinc in pigmented tissues. Julien and associates assessed in pigmented rats that zinc deficit leads to lipofuscin accumulation in the RPE and a decreased zinc mole fraction of melanosomes in the RPE [447]. This may indicate a link between zinc deficiency and altered autophagy, a condition typically observed in AMD pathogenesis. Aβ oligomers, which contribute to inflammation and oxidative stress in ARPE cells, also induce a block of autophagy. Sao et al. demonstrated that this condition can be reversed by treatment with clioquinol, a zinc ionophore, suggesting that manipulating lysosomal zinc levels may enhance the clearance of intracellular Aβ oligomers through increased autophagy [448]. In cardiac cells with hypoxia/reoxygenation injuries, Bian et al. showed that zinc can trigger mitophagy and increase ERK activity, collectively counteracting mitochondrial ROS excess [449].

Given the anti-inflammatory, antioxidant, and pro-autophagic activities of zinc, dietary supplementation may be hypothesized as a valuable tool in AMD prevention. Dedicated studies have reported that zinc supplementation in combination with the AREDS formula has the potential to slow AMD progression, while a higher intake of dietary zinc has beneficial effects on AMD incidence [450-453].

#### 4.2.4. Mitochondria-Targeting Therapeutic Molecules

PU-91, an FDA-approved mitochondrion-targeting drug for dyslipidemia management, was investigated by Nashine et al. in a cybrid model of AMD. The study demonstrated enhanced mitochondrial biogenesis and antioxidative potential by activating PGC-1α, along with improvements in inflammation and complement inhibitory activity [454]. The same research group expanded their investigation, revealing varied responses to PU-91 drug treatment among AMD cybrids with different mtDNA haplogroups [455]. A recent study by Salimiaghdam et al. found that PU-91 can improve cellular metabolism and reduce ROS formation in AMD cybrids. Additionally, a combination treatment of PU-91 plus quercetin induced upregulation of key factors like SOD2, IL-6, and BAX, suggesting potential benefits in terms of cellular metabolism and mitochondrial biogenesis in AMD cybrids, albeit leading to a senescent phenotype [456].

Mitochondrial-derived peptides, a new group of small molecules, have gained interest for their potential in countering AMD [457, 458]. Humanin, a component of this group, enhances mitochondrial biogenesis and mtDNA copy number, counters endoplasmic reticulum stress, and suppresses ROS-related cell death by STAT3 phosphorylation and caspase-3 activation [459, 460].An investigation on AMD cybrids revealed that humanin preserves AMD mitochondria, reducing pro-apoptosis agents and enhancing protection against amyloid-β-induced damage [461]. Solanki et al. tested a drug delivery system in ARPE-19 cells, demonstrating the effectiveness of humanin encapsulated in chitosan nanoparticles to prevent oxidative apoptosis, with desirable pharmacokinetic and biocompatible properties [462]. Nashine et al. also reported an anti-inflammatory effect of humanin on AMD RPE cybrid cells, decreasing levels of TNF-α, IFN-γ, and IL-1β [463].

SkQ1, also known as visomitin, is a mitochondrial-targeted antioxidant tested against various conditions, including hemorrhagic shock, toxic shock, and Alzheimer’s disease, with promising results [464-466]. In the eye, SkQ1 has been studied as an effective drug in vitro, countering ocular surface inflammation and improving corneal epithelial wound healing by enhancing cell proliferation and migration [467]. Importantly, SkQ1 showed promise in rodent models of AMD by suppressing atrophic changes in RPE, regulating expression of αB-crystallin, decreasing the mTOR signaling and reducing the level of Aβ deposition, and blocking the development of the neurodegenerative processes associated with AMD [468-471].

Nicotinamide mononucleotide (NMN) is a crucial intermediate of NAD^+^ required in the mitochondrial electron transport chain for the ATP production. Aging and oxidative stress can dramatically reduce physiological levels of NAD^+^. A study by Ebeling et al. has reported that supplementation with NMN improves mitochondrial function and ATP production in RPE cells obtained from AMD donors [472]. A recent investigation by Ren et al. demonstrated that NMN successfully treated senescent RPE cells induced by NaIO_3_, mitigating DNA damages and retaining mitochondrial function, while also exhibiting anti-inflammatory effects against subretinal cell infiltration [473].

Triphenylphosphonium (TPP), a mitochondrial-targeting molecule combined with vitamin B3 (niacin) in TPP-niacin, has been assessed to alleviate ROS-related mitochondrial dysfunction. This compound upregulated mitochondrial-associated genes, improving cell viability and augmenting antioxidant enzyme activity in ROS-exposed ARPE-19 cells [474]. Consistent with these findings, Nguyen and associates confirmed the effectiveness of TPP-Niacin in contrasting oxidative stress and mitochondrial dysfunction *in vitro* [475].

Elamipretide, a mitochondria-targeted tetrapeptide, stabilizes cardiolipin and increases cellular ATP synthesis while decreasing mitochondrion-induced ROS [476]. Two phase 1 clinical trials have assessed the efficacy of elamipretide in intermediate AMD [477], and in dry AMD/non-central GA [478], displaying encouraging results. A Phase 2b trial is ongoing to further assess elamipretide in dry AMD.

#### 4.2.5. Programmed Cell Death Inhibitors

In recent years, a growing number of preclinical studies have exhibited promise by demonstrating the effectiveness of specific molecules in blocking cell death pathways in RPE cells, presenting themselves as potential therapeutic strategies for AMD.

There is no universally recognized single cell death pathway predominant in AMD based on existing literature. Different stressors can implicate diverse cell death transductions and molecular signaling [479]. Treatment strategies should consider addressing issues related to the intersection of different cell death pathways to comprehensively counteract cell loss in multiple transductions. In this context, a comparative study on cell death mechanisms in RPE cells suggested that the necroptosis inhibitor, necrostatin-1 (Nec-1), may be a possible therapeutic agent for GA. It showed inhibition of RIPK1/RIPK3 activation and lipid ROS accumulation in both necroptosis and ferroptosis pathways [480]. A prior study by Jang and associates demonstrated in *in vivo* models of dry AMD that a RIPK1 inhibitor possesses a protective effect on RPE cells through fundus evaluation and electroretinogram analyses, assessing effective retinal penetration and prevention of retinal degeneration in dry AMD [481].

Hwang and Chung showed that sulfasalazine can decrease tamoxifen-induced ROS overabundance, along with the expression of tamoxifen-induced pyroptosis-related genes, IL-1β, NLRP3, and procaspase-1. Additionally, there was a downregulation of tamoxifen-induced AMD-related genes, such as CFH and TLR2 [482]. Tripartite motif (TRIM) 31, a component of the TRIM family functioning as an E3 ubiquitin ligase, is relevant in the posttranslational modification of proteins known as ubiquitination [483]. Huang et al. demonstrated in ARPE-19 cells that TRIM31 can counteract Ox-LDL-related pyroptosis through ubiquitination of NLRP3 [484]. Sun and associates reported that baicalein, a constituent of the plant *Scutellaria baicalensis*, mitigates Aβ-related pyroptosis in human RPE cells via negative crosstalk of miR-223/NLRP3 inflammasome signaling [485]. Another preclinical investigation on ARPE-19 cells described that *Lycium barbarum* polysaccharides can disrupt the Aβ-oligomerization, thereby leading to a lack of Aβ-induced pyroptosis activation [486]. A recent study reported that lutein shows promise in ARPE-19 cells to combat pyroptotic activation via downregulation of pyroptosis-related genes such as, for example, the pivotal caspase 1 [487].

Antagonizing ferroptosis may be effective in alleviating neurodegeneration in the auditory cortex [488]. In this context, an interesting work by Tang and colleagues assessed that targeting the HO-1-associated ferroptosis may be a valuable strategy against AMD. It highlights the use of a HO-1 inhibitor, ZnPP, in significantly blocking RPE ferroptosis, leading to a substantial improvement in retinal structure and visual function in mice [489].

An insightful review by Zacks and colleagues examined the possibility of targeting Fas receptor to counteract programmed cell death in GA in multiple ways [490]. The interaction between Fas ligand and its receptor is responsible not only for caspase activation but also of pro-inflammatory reactivity, via cytokine and chemokine production [491, 492]. Hence, the Fas receptor may be a potential suitable molecular target to counteract disease progression in AMD. In this respect, a dedicated preclinical investigation in a rodent model exposed to oxidative stress assessed that a small peptide antagonist of the Fas receptor, Met12, significantly decreased the activation of the Fas-mediated death signaling, such as apoptosis and necroptosis, preserving RPE cell and photoreceptors survival and reducing the inflammatory response [493].

## 5. Concluding Marks and Future Perspectives

Due to its vital functions in the visual system, the macula demands an exceptionally high amount of oxygen. Additionally, the central retina is perpetually and intensely exposed to high UV irradiation as part of its physiological activity. Consequently, this region is highly susceptible to oxidative stress. Considering the multifactorial nature of AMD, factors such as aging, specific genetic backgrounds, light exposure, or cigarette smoking can lead to a pro-inflammatory and pro-oxidative imbalance in RPE, contributing to the onset of the disease. Late AMD, particularly the prevalent variant of slowly progressive geographic atrophy (GA), often results in irreversible visual loss, posing a significant concern for patients and public health. The recent FDA approval of complement inhibitors addresses the unmet medical need for effective medications to counteract the growth of atrophic lesions in GA. Research progress, through experimental and clinical investigations, may introduce innovative treatment strategies, offering new possibilities for combating AMD in various modalities. The foundation for such endeavors lies in a thorough understanding of the intricate disease pathophysiology, focusing on identifying the most suitable molecular targets. In this regard, our review comprehensively summarizes recent advancements and new insights into the understanding of the disease etiopathogenesis, with a specific emphasis on molecular pathways related to aging, oxidative stress, inflammation, mitochondrial dysfunction, and complement anomalies, and their complex interrelation in AMD. Encouraging and promising molecules examined in preclinical studies and clinical trials are also presented. Given the multifaceted pathogenesis of the disease, it seems reasonable to invest efforts in testing molecules with different targets, potentially suitable for multi-targeted therapy. Such treatments may effectively counteract disease progression by antagonizing different signaling pathways, proving efficacy in diverse stages. An individualized multi-targeting treatment approach is likely to become the future in therapeutic options for AMD. The FDA licenses of pegcetacoplan and avacincaptad pegol align with this direction, specifically authorized for the management of GA, offering new prospects and raising expectations in this field with the introduction of novel molecular targets. However, future research should provide evidence regarding the cost-effectiveness and duration of therapy for these new drugs. Testing antioxidant supplements as an effective tool to prevent disease onset is also crucial, given the irreversible progression of the disease, often leading to GA. Large clinical trials in this prospect offer an opportunity to advance the fight against this disabling disease, providing essential data on pharmacokinetics and safety profiles for human use, crucial for ensuring satisfactory levels of tolerance and efficacy.
